# Broad and potent neutralizing human antibodies to tick-borne flaviviruses protect mice from disease

**DOI:** 10.1084/jem.20210236

**Published:** 2021-04-08

**Authors:** Marianna Agudelo, Martin Palus, Jennifer R. Keeffe, Filippo Bianchini, Pavel Svoboda, Jiří Salát, Avery Peace, Anna Gazumyan, Melissa Cipolla, Tania Kapoor, Francesca Guidetti, Kai-Hui Yao, Jana Elsterová, Dana Teislerová, Aleš Chrdle, Václav Hönig, Thiago Oliveira, Anthony P. West, Yu E. Lee, Charles M. Rice, Margaret R. MacDonald, Pamela J. Bjorkman, Daniel Růžek, Davide F. Robbiani, Michel C. Nussenzweig

**Affiliations:** 1 Laboratory of Molecular Immunology, The Rockefeller University, New York, NY; 2 Institute of Parasitology, Biology Centre of the Czech Academy of Sciences, České Budějovice, Czech Republic; 3 Veterinary Research Institute, Brno, Czech Republic; 4 Division of Biology and Biological Engineering, California Institute of Technology, Pasadena, CA; 5 Institute for Research in Biomedicine, Università della Svizzera italiana, Bellinzona, Switzerland; 6 Department of Pharmacology and Pharmacy, Faculty of Veterinary Medicine, University of Veterinary and Pharmaceutical Sciences Brno, Brno, Czech Republic; 7 Laboratory of Virology and Infectious Disease, The Rockefeller University, New York, NY; 8 Hospital České Budějovice, České Budějovice, Czech Republic; 9 Faculty of Social and Health Sciences, University of South Bohemia, České Budějovice, Czech Republic; 10 Royal Liverpool University Hospital, Liverpool, UK; 11 Howard Hughes Medical Institute, The Rockefeller University, New York, NY

## Abstract

Tick-borne encephalitis virus (TBEV) is an emerging human pathogen that causes potentially fatal disease with no specific treatment. Mouse monoclonal antibodies are protective against TBEV, but little is known about the human antibody response to infection. Here, we report on the human neutralizing antibody response to TBEV in a cohort of infected and vaccinated individuals. Expanded clones of memory B cells expressed closely related anti-envelope domain III (EDIII) antibodies in both groups of volunteers. However, the most potent neutralizing antibodies, with IC_50_s below 1 ng/ml, were found only in individuals who recovered from natural infection. These antibodies also neutralized other tick-borne flaviviruses, including Langat, louping ill, Omsk hemorrhagic fever, Kyasanur forest disease, and Powassan viruses. Structural analysis revealed a conserved epitope near the lateral ridge of EDIII adjoining the EDI–EDIII hinge region. Prophylactic or early therapeutic antibody administration was effective at low doses in mice that were lethally infected with TBEV.

## Teaser

Agudelo et al. discover expanded clones of similar antibodies from the memory B cells of tick-borne encephalitis virus–infected and vaccinated donors; however, the most potent and broadly neutralizing antibodies derive from donors recovering from natural infection.

## Introduction

Tick-borne flaviviruses are responsible for a series of emerging infectious diseases including fatal encephalitis. Like other flaviviruses, the tick-borne encephalitis virus (TBEV) envelope protein (E) is composed of three structural domains (envelope domains I–III [EDI–EDIII]; Füzik et al., 2018; Pulkkinen et al., 2018). Mouse monoclonal antibodies against EDIII, an Ig-like domain that mediates host cell attachment, are potent neutralizers of TBEV (Baykov et al., 2014; Füzik et al., 2018; Levanov et al., 2010; Matveev et al., 2020; Phillpotts et al., 1985; Rey et al., 1995; Yang et al., 2019).

TBEV is one of the six flaviviruses transmitted by ticks causing human disease (Gould and Solomon, 2008; Kuno et al., 1998; LaSala and Holbrook, 2010). These include Omsk hemorrhagic fever virus (OHFV) in Russia; Kyasanur forest disease virus (KFDV) in India; Alkhurma virus in Saudi Arabia; louping ill virus (LIV) in the United Kingdom, Ireland, Norway, Denmark, and Russia; and Powassan virus in the United States and Canada. Upwards of 10,000 TBEV cases per year are reported, with a trend for increased incidence in recent years and emergence of the disease in new geographic regions (Beauté et al., 2018; Girl et al., 2020; Kollaritsch et al., 2011; Morens and Fauci, 2020; Smura et al., 2019; Süss et al., 2006; Yoshii, 2019; Zeman and Bene, 2004).

The bite of an infected tick, or the consumption of unpasteurized milk from infected animals, causes a biphasic illness, which begins with a period of influenza-like symptoms followed by the development of neurological disease (tick-borne encephalitis [TBE]). There is no specific therapy for TBE, and treatment is limited to supportive care. For those individuals who survive, long-term sequelae are common (Bogovič et al., 2018b; Caini et al., 2012; Cisak et al., 2010; Donoso-Mantke et al., 2011; Holzmann, 2003; Holzmann et al., 2009; Kaiser, 2008).

Although TBEV vaccines are available, immunity requires regular boosting, and vaccination is less effective in the young and elderly. Vaccination requires administration of three separate doses spaced over up to 2 yr, with booster doses recommended at intervals of 3–5 yr (World Health Organization, 2019). Breakthrough TBEV infection occurs despite vaccination (Dobler et al., 2020; Lotrič-Furlan et al., 2017).

Recovered individuals can produce potent serologic neutralizing activity against TBEV, and the serum can be cross-reactive against other tick-borne flaviviruses, but the nature of the antibodies that mediate these effects is not known (Calisher et al., 1989; Mansfield et al., 2011). Postexposure prophylaxis with hyperimmune plasma Ig from vaccinated donors provides protection if administered within 3 d of an infected tick bite (Kreil et al., 1998; Pen’evskaia and Rudakov, 2010). However, this practice was discontinued outside of Russia due to anecdotal evidence of adverse events in children and the concern of possible antibody-dependent enhancement of disease (Halstead, 2014; Phillpotts et al., 1985; Ruzek et al., 2019).

Here, we report on the molecular properties of human anti-TBEV antibodies with exceptional neutralizing potency and breadth that are effective in protection and early therapy in mice.

## Results

### Serological responses in a TBEV-infected cohort

**Figure 1.**
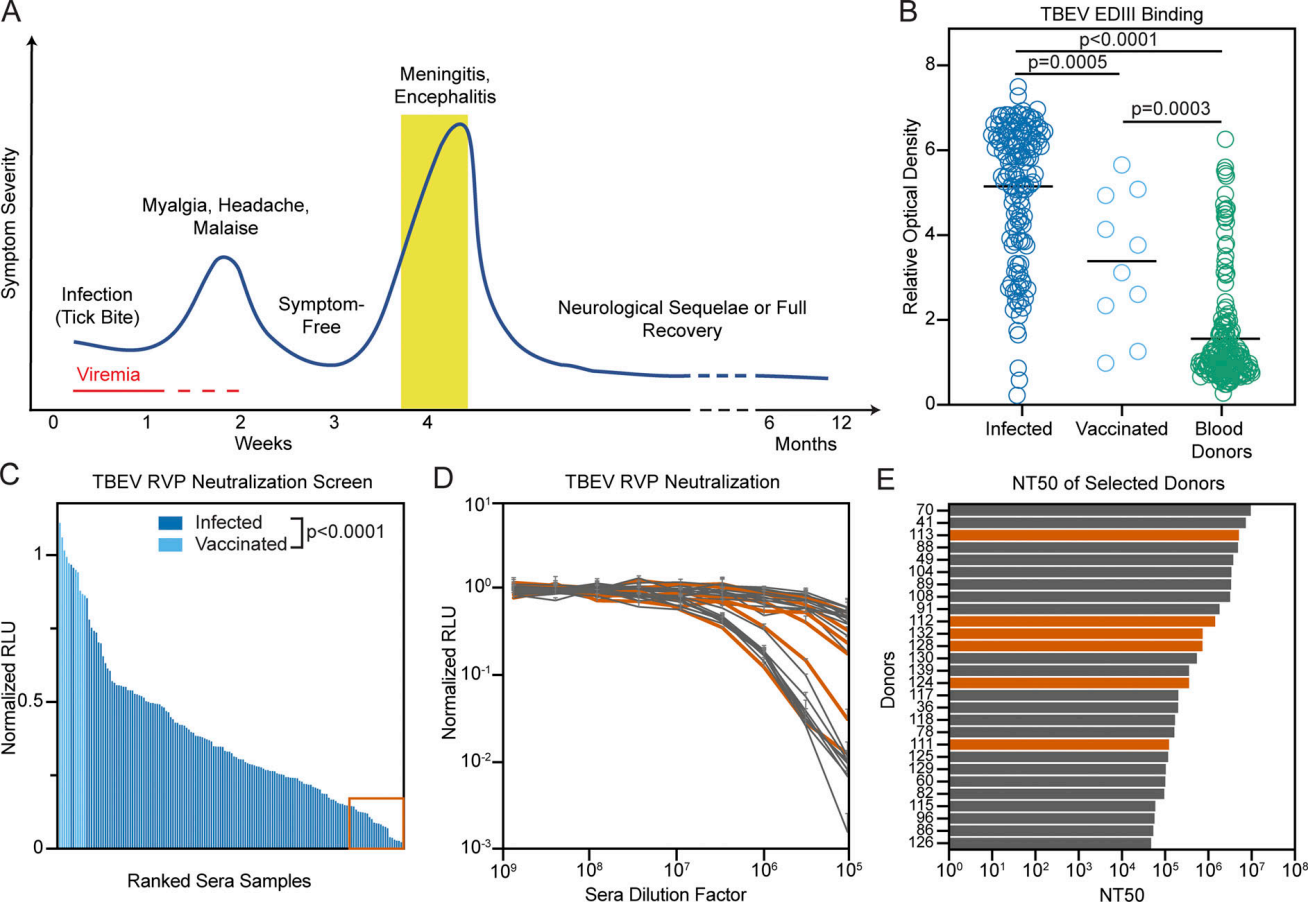
**Screening individuals for TBEV antibodies.** **(A)** Diagrammatic representation of the clinical course of TBE. The approximate time of serum collection is shown in yellow. **(B)** TBEV EDIII IgG ELISA. Graph shows optical density measurement (y axis) relative to a negative control serum for samples from 141 TBEV-infected individuals, 10 TBEV vaccinees, and 168 random blood donors (1:500 dilution) measured in singlicate. P = 0.0005 for infected versus vaccinees; P < 0.0001 for infected versus blood donors; P = 0.0003 for vaccinees versus blood donors; calculated by one-way ANOVA followed by Tukey’s test. Horizontal lines indicate the mean. **(C)** TBEV RVP neutralization screening. Graph shows ranked serum neutralizing activity (1:600,000 dilution) against TBEV RVPs (average of duplicate wells) relative to no serum control. The orange box (bottom right) indicates the 28 best neutralizers of 141 TBEV-infected individuals and 10 TBEV vaccinees tested. P < 0.0001; calculated using two-tailed Mann–Whitney test. **(D)** TBEV RVP neutralization curves. Plot shows representative neutralization curves for each of the 28 most potent sera from C. Representative of two experiments, each performed in triplicate. Error bars indicate standard deviation. **(E)** Ranked NT_50_s for the top 28 individuals. Average of two independent experiments. In D and E, orange indicates the donors of PBMCs for antibody cloning. Related to Fig. S1 and Table S1. RLU, relative light units.

**Figure S1.**
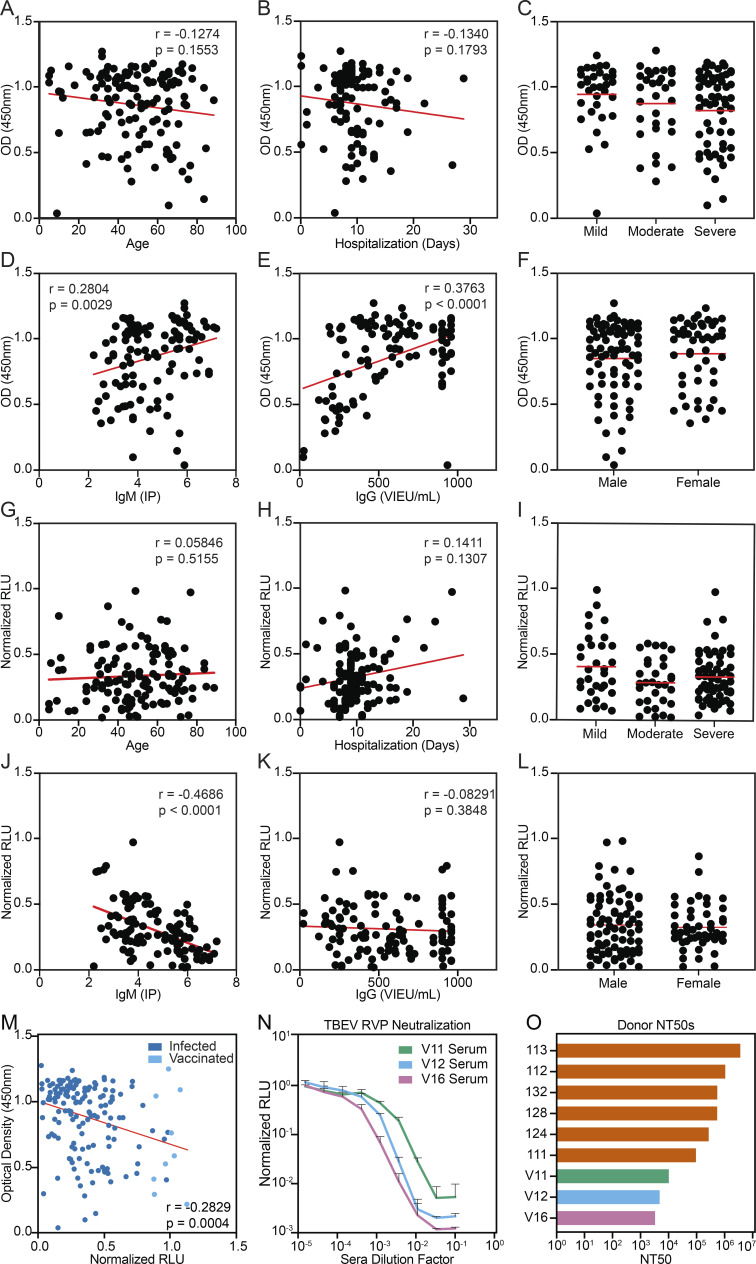
**Clinical correlations and serum neutralization in vaccinees.** **(A–F)** Serum TBEV EDIII ELISA data (IgG) from Fig. 1 B plotted against demographic and available clinical information. **(G–L)** Serum TBEV RVP neutralization data from Fig. 1 C plotted against demographic and available clinical information. A and G show age (no significance), B and H show length of hospitalization (no significance), C and I show severity of disease (no significant differences), D and J show IgM titers (IP) measured at the time of hospitalization (P = 0.0029 and P < 0.0001, respectively); E and K show IgG titers (Vienna units/ml) measured at the time of hospitalization (P < 0.0001 and no significance, respectively), and F and L show patient gender (no significant differences). Statistical significance was calculated for A, B, D, E, G, H, J, and K using two-tailed Spearman's tests; for L and F using Mann–Whitney tests; and for C and I using one-way ANOVA with Tukey’s test. **(M)** Correlation between serum TBEV EDIII ELISA (IgG) and RVP neutralization data. **(N)** TBEV RVP neutralization curves with sera from vaccinated PBMC donors. Representative of two experiments, each performed in triplicate. Data are presented as mean with standard deviation. **(O)** Summary of serum NT_50_s for all infected and vaccinated PBMC donors. Related to Fig. 1 and Table S1. RLU, relative light units.

We analyzed sera from 141 individuals hospitalized with TBE during the 2011 and 2018 outbreaks in the Czech Republic (see Materials and methods). Samples were obtained at the time of hospitalization during the encephalitic phase of disease (Fig. 1 A; Holzmann, 2003). In agreement with previous reports (Bogovič et al., 2018a; Bogovic and Strle, 2015), the cohort was characterized by higher incidence in males (61.1%) and older individuals (mean age, 49 yr; Table S1). Control sera were also obtained from 168 randomly selected blood bank donors and 10 individuals vaccinated against TBEV (Table S1). All sera were screened by ELISA at a dilution of 1:500 for the presence of IgG antibodies binding to the EDIII of TBEV (Fig. 1 B). The signal in infected individuals was significantly higher than in the vaccinated and blood donor groups (P = 0.0005 and P < 0.0001 by ANOVA using Tukey’s correction, respectively; Fig. 1 B). There was no correlation between TBEV EDIII ELISA reactivity and age, duration of hospitalization, severity of infection, or gender of patient, but serum EDIII binding did correlate positively with patient IgM (P = 0.0029) and IgG (P < 0.0001) levels at the time of hospitalization (Fig. S1, A–F).

To evaluate serum neutralizing activity, samples obtained from recovered and vaccinated individuals were screened at a 1:6 × 10^5^ dilution for neutralization using luciferase-expressing TBEV reporter virus particles (RVPs; see Materials and methods; Pierson et al., 2006). Neutralizing activity ranged from complete to undetectable and was significantly lower in vaccinees (P < 0.0001; Fig. 1 C). Neutralization correlated positively with patient IgM levels at the time of hospitalization (P < 0.0001) and EDIII binding in ELISA (P = 0.0004); otherwise, there was no correlation with demographic or clinical data (Fig. S1, G–M). The half-maximal neutralizing titers (NT_50_) for the top 28 infected individuals varied from 0.37 to 6.7 × 10^6^ (Fig. 1, D and E). In contrast, vaccinees showed NT_50_s of 0.32–1.0 × 10^4^ (Fig. S1, N and O). We conclude that individuals hospitalized for TBEV infection show a broad distribution of EDIII binding and neutralizing activity that is generally higher than the vaccinees in this cohort.

### B cell memory convergence on specific antibody genes

**Figure 2.**
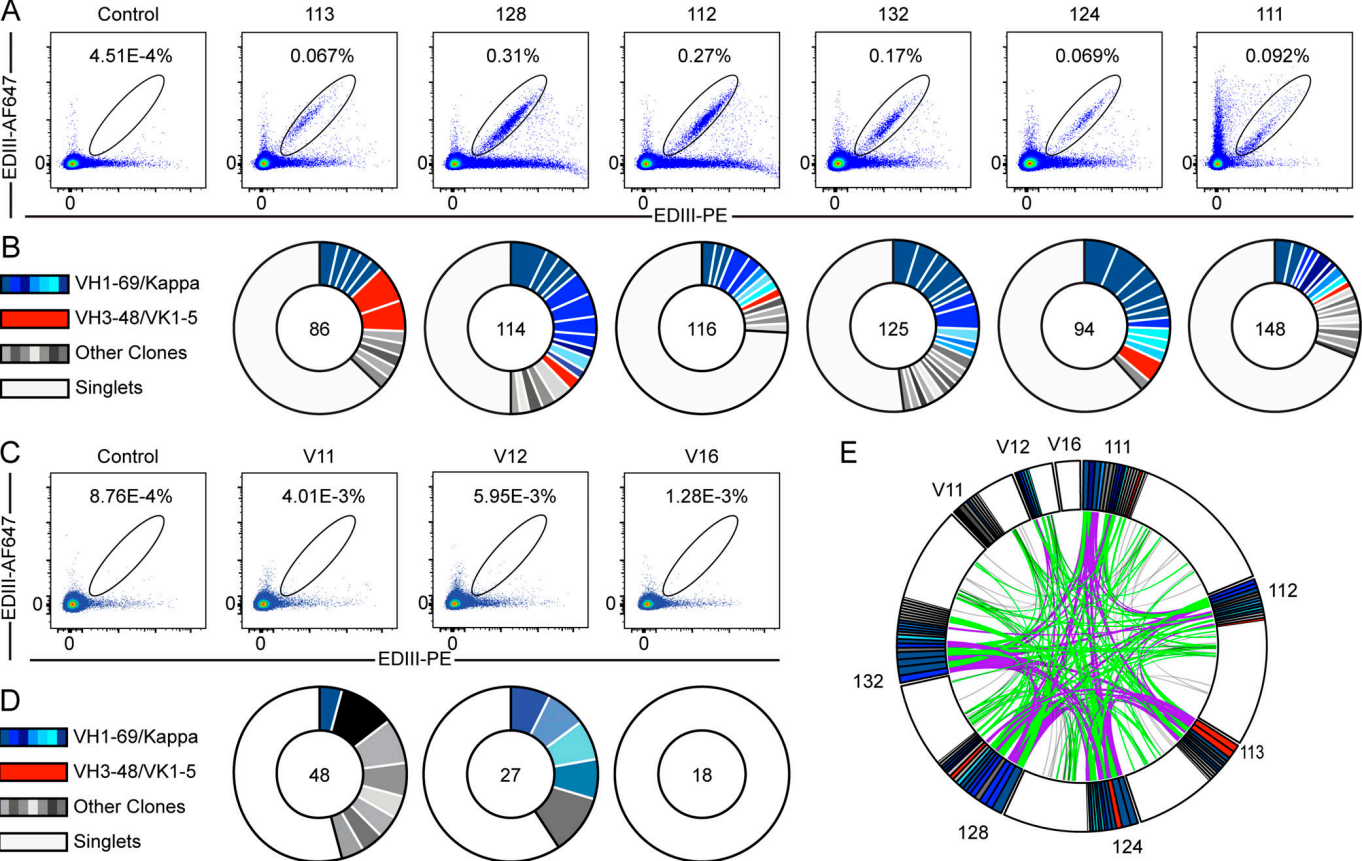
**Anti-TBEV antibodies from infected and vaccinated individuals.** **(A)** Identification of TBEV-specific B cells from infected donors. Representative flow cytometry plots showing B cells binding to AF647- and PE-labeled TBEV EDIII in one control and six TBEV-infected donors. Numbers indicate percentage of double-positive B cells. The gating strategy is shown in Fig. S2 A. **(B)** Clonal analysis of antibody sequences. Pie charts show the distribution of antibody sequences from infected donors. The number in the center represents the total number of antibody sequences obtained. Colored or gray pie slices correspond to clonally related sequences, with the size of the slice proportional to the number of sequences. All blue slices are IGVH1–69, and all red slices are IGVH3–48/IGVK1–5. White slices correspond to antibody sequences that are not part of a clone (singlets). **(C** **and D)** Same as in A and B, but for one healthy control and three vaccinated donors. **(E)** Antibody sequence relatedness. Circos plot shows sequences from all donors, with color-coding as in B and D. Connecting lines indicate antibodies that share IGH and IGL V and J genes. Purple, green, and gray lines connect related clones to each other, clones to singlets, and singlets to singlets, respectively. Related to Figs. S2 and S3, Table S2, and Table S3.

**Figure S2.**
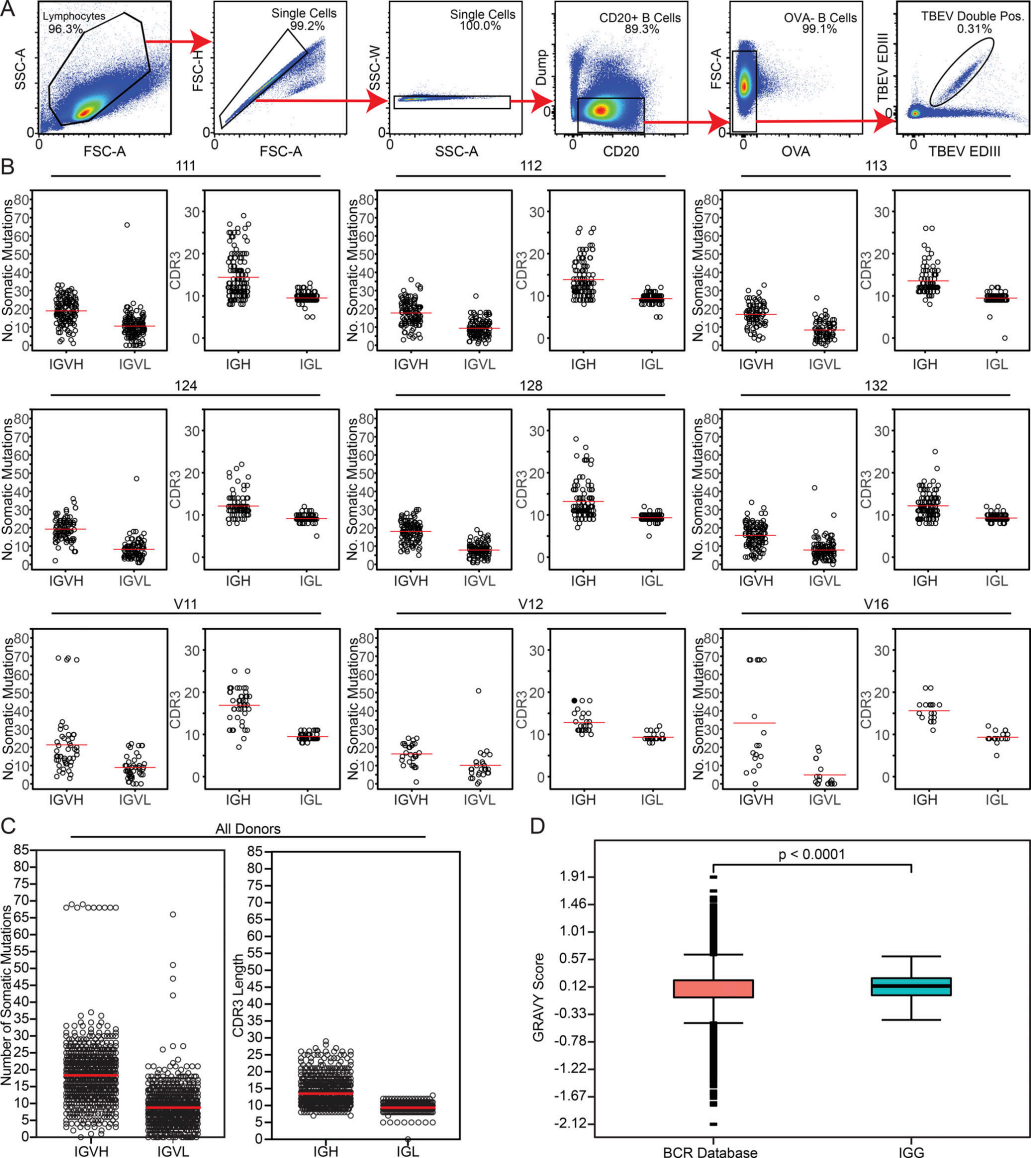
**Sorting strategy and antibody sequence analysis.** **(A)** Sorting strategy. Forward and side scatter (FSC and SSC, respectively) were used to gate on single lymphocytes. Dump channel included CD3, CD8, CD14, CD16, and a viability dye. CD20^+^ B cells that failed to bind OVA (OVA^−^) but did bind to the TBEV EDIII bait coupled with both PE and AF647 fluorophores were purified. **(B)** For each donor, the number of V gene somatic nucleotide mutations is shown on the left and the amino acid length of the CDR3 is shown on the right. **(C)** As in B, but for all donors combined. For B and C, horizontal red lines indicate the mean. **(D)** Distribution of hydrophobicity GRAVY scores at the IGH CDR3 of antibodies from all donors combined and compared with human repertoire (Briney et al., 2019). P < 0.0001 was determined using the Wilcoxon nonparametric test. Related to Fig. 2, Table S2, and Table S3.

To characterize the anti-TBEV antibodies, we purified B cells that bind to TBEV EDIII from peripheral blood of six infected individuals (orange in Fig. 1, D and E) and three vaccinees (see Materials and methods; Fig. 2, A–D; and Fig. S2 A). The frequency of TBEV EDIII–specific B cells was higher in the infected group (0.067–0.31%) compared with vaccinees (1.28 × 10^−3^–5.95 × 10^−3^%). In total, 776 IgG antibody heavy and light chain gene pairs were amplified by RT-PCR and sequenced (see Materials and methods; Fig. 2, B and D; and Table S2). The average somatic hypermutation in Ig heavy and light chain genes (Ig variable heavy gene [IGVH], Ig variable kappa gene [IVGK], and Ig variable light gene [IGVL]) was 18 and 9 nt, respectively, complementarity-determining region 3 (CDR3) length was normal (mean CDRH3 length of 13.5 and mean CDRL3 length of 9.4), and hydrophobicity was slightly increased compared with control (P < 0.0001; Fig. S2, B–D; Briney et al., 2019; Rock et al., 1994). As with other viral pathogens, including HIV-1, Zika, hepatitis B, and SARS-CoV-2 (Robbiani et al., 2017; Robbiani et al., 2020; Scheid et al., 2011; Wang et al., 2020; West et al., 2012), many of the sequences were found in expanded B cell clones (37.9%; Fig. 2, B and D).

**Figure S3.**
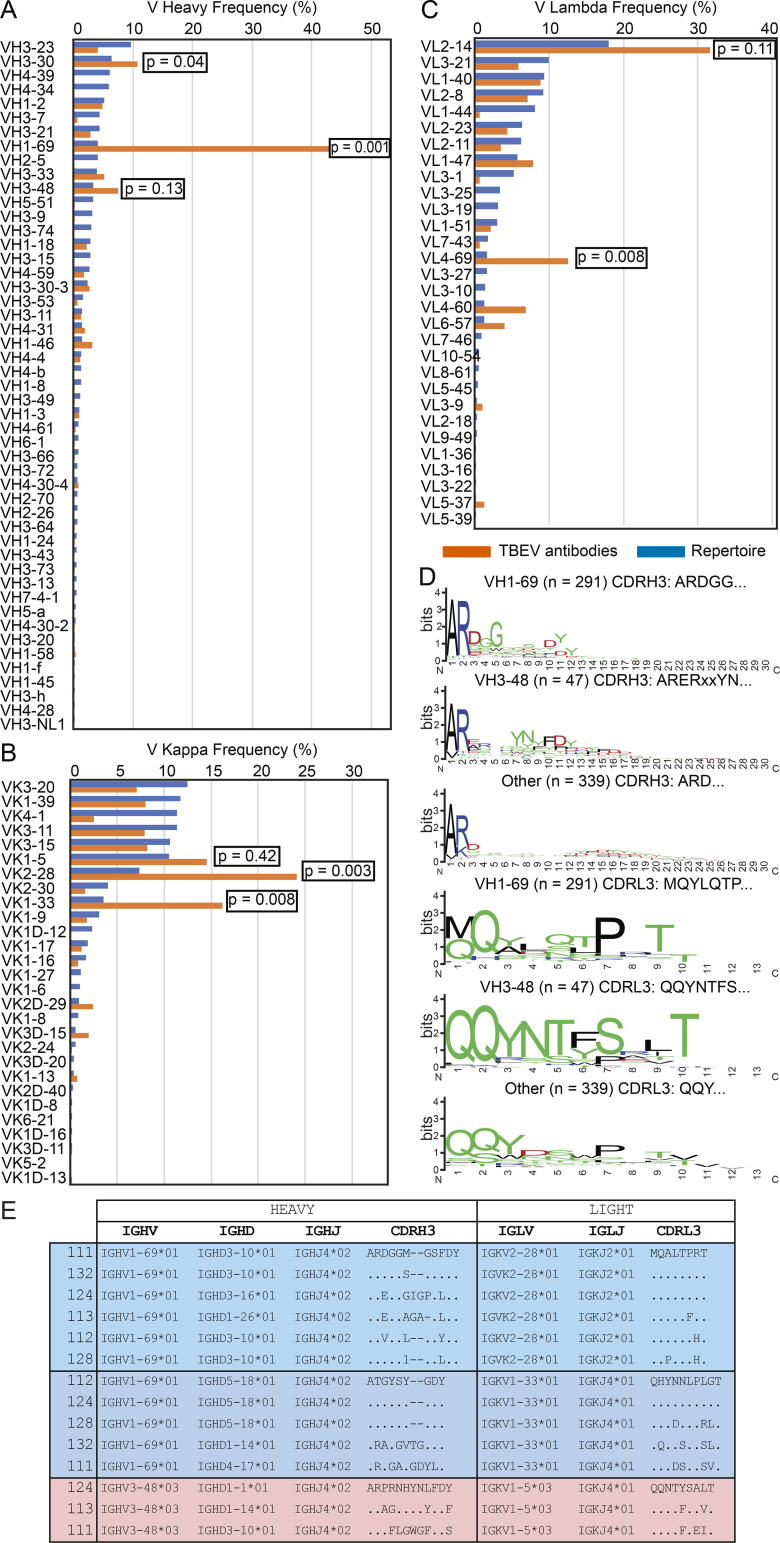
**Antibody V gene frequency and CDR3 sequences.** **(A)** Bar graph showing the frequency of V heavy gene usage in TBEV antibodies from infected donors compared with human repertoire (Rubelt et al., 2012). **(B** **and** **C)** As in A, but for V kappa and V lambda genes. In A–C, orange indicates anti-TBEV antibodies isolated in this study, while blue indicates control repertoire; P values were calculated using a two-tailed *t* test with unequal variances. **(D)** Sequence logos for antibody CDR3s from infected donors generated by WebLogo. The height of the stack indicates the sequence conservation at a given position, while the height of letters within the stack indicates the relative frequency of each amino acid at that position. **(E)** Examples of highly similar antibody sequences found in multiple donors. Related to Fig. 2, Table S2, and Table S3.

Sequence analysis revealed antibodies with similar features within and between individuals (Fig. 2, B, D, and E; Table S2; and Table S3). For example, VH1–69 and VH3–48 accounted for 59.2% and 7.5% of all clonal sequences, respectively (shades of blue and red in Fig. 2, B and D). In addition, related sequences containing these VH genes were found in multiple donors (purple lines in Fig. 2 E). Usage of VH1–69, VH3–30, VK2–28, VK1–33, and VL4–69 genes in infected donors was significantly overrepresented (P < 0.01); VH3–48, VK1–5, and VL2–14 genes were also enriched, although not significantly (Fig. S3, A–C). In some cases, including clonally expanded IGVH1–69/Ig variable kappa gene 2–28 (IGVK2–28) and IGVH3–48/IGVK1–5 antibodies, sequence similarities between individual donors extended to the IGH and IGL CDR3s (Fig. S2, D and E; and Table S3). We conclude that the memory B cell response to the TBEV EDIII converges toward specific antibody genes.

### Potent and broadly cross-reactive anti-TBEV antibodies

**Figure 3.**
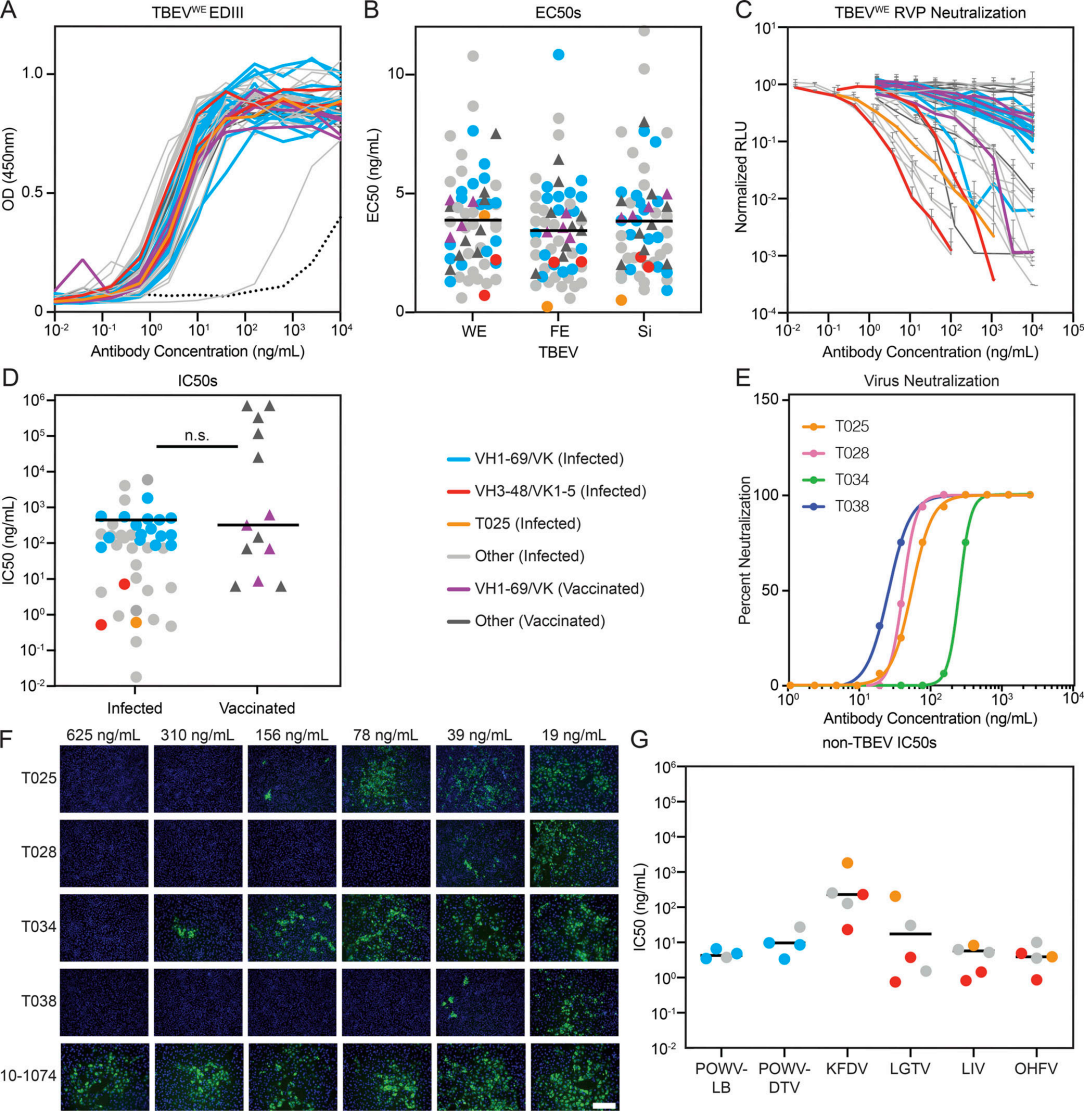
**Identification of potent and broadly cross-reactive monoclonal antibodies.** **(A)** TBEV^WE^ EDIII ELISA binding curves for 46 and 13 monoclonals from infected and vaccinated individuals, respectively. Data are representative of two experiments performed in singlicate. Dotted line is 10–1074 isotype control. **(B)** Dot plot summarizing average EC_50_ values for the antibodies in A to each of three TBEV subtype EDIIIs: TBEV^WE^, TBEV^FE^, and TBEV^Si^. Average of two experiments. The horizontal lines indicate the mean value. **(C)** RVP neutralization curves for the antibodies in A normalized to no antibody control. Data are representative of two experiments, each performed in triplicate. Error bars indicate standard deviation. **(D)** Dot plot summarizing the average IC_50_ for TBEV^WE^ RVP neutralization by the antibodies as in C. Horizontal line indicates the mean IC_50_. No statistical difference was found by two-tailed Mann–Whitney test. **(E** **and** **F)** TBEV neutralization in vitro. In E, curves represent virus neutralization by serially diluted antibodies. Representative of two independent experiments performed in octuplicate. In F, representative immunofluorescence microscopy images of PS cells infected in the presence of the indicated antibodies are shown. Green is viral antigen, and blue is cell nuclei. Scale bar indicates 200 µm. **(G)** Cross-neutralization by anti-TBEV antibodies. Graph shows IC_50_s for selected antibodies against RVPs corresponding to POWV-LB, POWV-DTV, KFDV, LGTV, LIV, and OHFV. Average of two independent experiments performed in triplicate. Horizontal line indicates the mean IC_50_. In A–D and G, blue and red indicate infected donor-derived IGVH1–69/kappa and IGVH3-48/IGVK1–5 antibodies, while purple indicates IGHV1–69/kappa antibodies from vaccinated individuals. Antibody T025 is shown in orange. In B, D, and G, circles and triangles correspond to antibodies derived from infected or vaccinated donors, respectively. Related to Fig. S4, Table S4, and Table S5.

**Figure S4.**
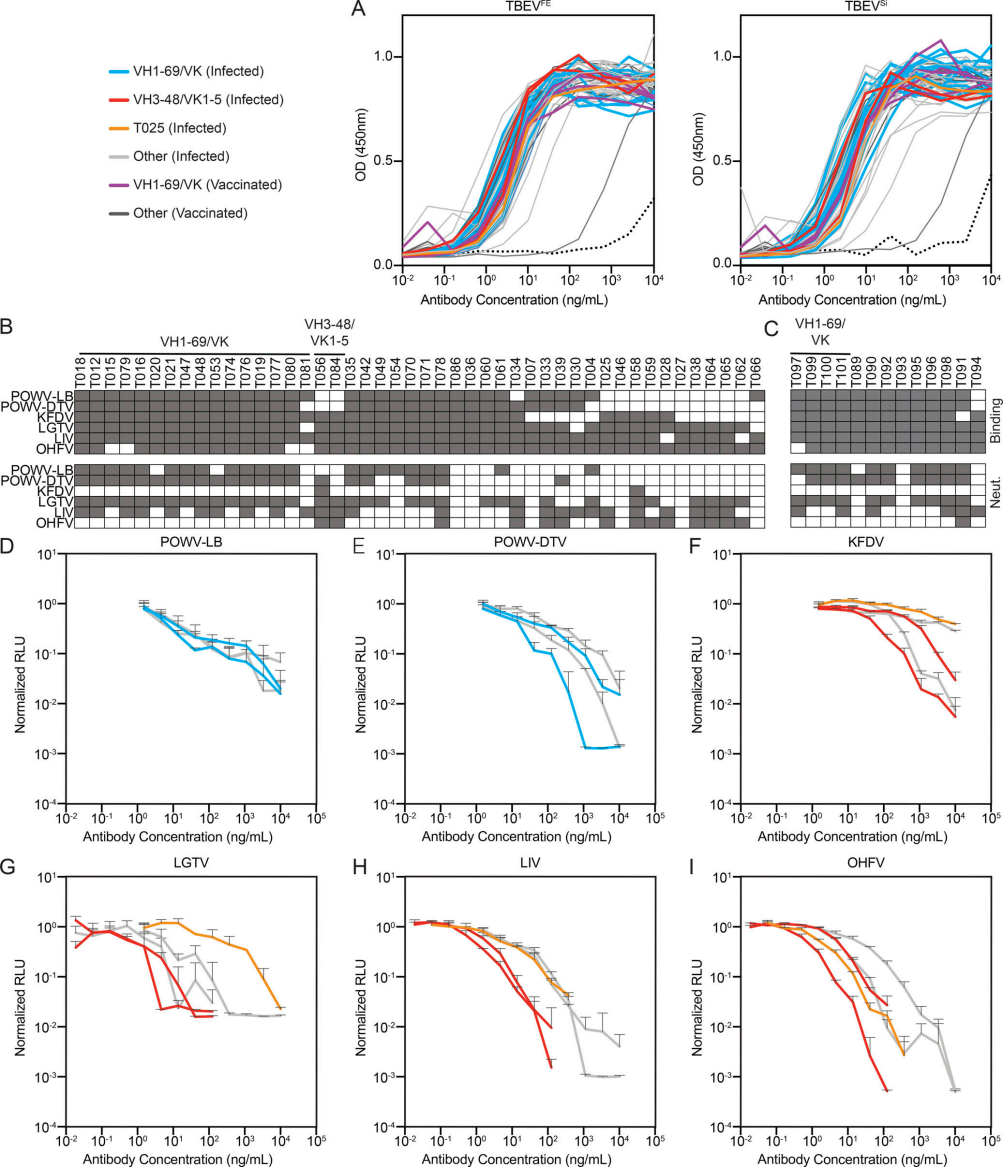
**Antibody binding and neutralization.** **(A)** ELISA binding curves to TBEV^FE^ and TBEV^Si^ EDIII for the 59 antibodies. Data are representative of two experiments. **(B)** Top panel shows screening for infected donor antibodies binding to a panel of tick-borne flavivirus EDIIIs, including POWV-LB, POWV-DTV, KFDV, LGTV, LIV, and OHFV. Antibodies were screened in duplicate at 1 µg/ml. Bottom panel shows screening for neutralization against RVPs corresponding to the same panel of tick-borne flaviviruses. Infected donor antibodies were screened in triplicate at 1 µg/ml. **(C)** Screening for vaccine antibodies binding and neutralization against a panel of tick-borne flavivirus EDIIIs and RVPs as in B. In B and C, gray indicates binding or neutralization over control. **(D–I)** Neutralization curves of selected antibodies against tick-borne flavivirus RVPs other than TBEV. Representative of two experiments in triplicate. Error bars indicate standard deviation. Related to Fig. 3, Table S4, and Table S5.

59 antibodies (46 from convalescent and 13 from vaccinated donors; Table S4) were cloned, recombinantly expressed, and tested in ELISA for binding to EDIII proteins corresponding to all three TBEV subtypes: Western European (TBEV^WE^), Far Eastern (TBEV^FE^), and Siberian (TBEV^Si^; Fig. 3 A, Fig. S4 A, and Table S5). All but 1 of the 59 antibodies bound to all three EDIIIs with similar half-maximal effective concentrations (EC_50_) ranging from 0.2 to 12 ng/ml (Fig. 3 B and Table S5).

When tested for neutralizing activity, 43 out of 46 antibodies obtained from infected donors neutralized TBEV RVPs with half-maximal inhibitory concentrations (IC_50_s) as low as 0.02 ng/ml (Fig. 3, C and D; and Table S5). In contrast, the best antibody obtained from vaccinated donors was over two orders of magnitude less potent (IC_50_ of 8.3 ng/ml). Seven antibodies, all isolated from infected donors, were potent neutralizers of TBEV RVPs with IC_50_s below 1 ng/ml (Fig. 3 D). Four of these antibodies were also evaluated for neutralization of authentic TBEV (Fig. 3, E and F). All four antibodies showed potent activity, with IC_50_s ranging from 35.9 to 268.8 ng/ml (Table S5).

To determine whether the TBEV antibodies cross-react with related viruses, we screened them at a single concentration (1 µg/ml) for binding to the EDIIIs of Langat virus (LGTV), LIV, OHFV, KFDV, and Powassan lineage I and II viruses (Powassan-LB virus [POWV-LB] and Powassan deer tick virus [POWV-DTV]; see Materials and methods and Fig. S4, B and C). Broad cross-reactivity was observed for many of the antibodies tested (Fig. S4, B and C). To determine whether the antibodies are also broadly neutralizing, we screened them against RVPs corresponding to the same panel of tick-borne viruses. When tested at a concentration of 1 µg/ml, most of the IGHV1–69 antibodies neutralized LGTV, LIV, POWV-LB, and POWV-DTV, and one of the IGVH3–48/IGVK1–5 antibodies neutralized all RVPs except POWV-LB (Fig. S4, B and C). IC_50_s against the flavivirus RVP panel were <10 ng/ml for several of the cross-reactive antibodies (Fig. 3 G; Fig. S4, D–I; and Table S5). For example, an IGVH3–48/IGVK1–5 antibody, T056, is a potent neutralizer of LGTV, LIV, and OHFV, with IC_50_ values ≤1 ng/ml. We conclude that some TBEV neutralizing antibodies are broadly active against tick-borne flaviviruses.

### Antibody T025 binds to the EDIII lateral ridge

**Figure 4.**
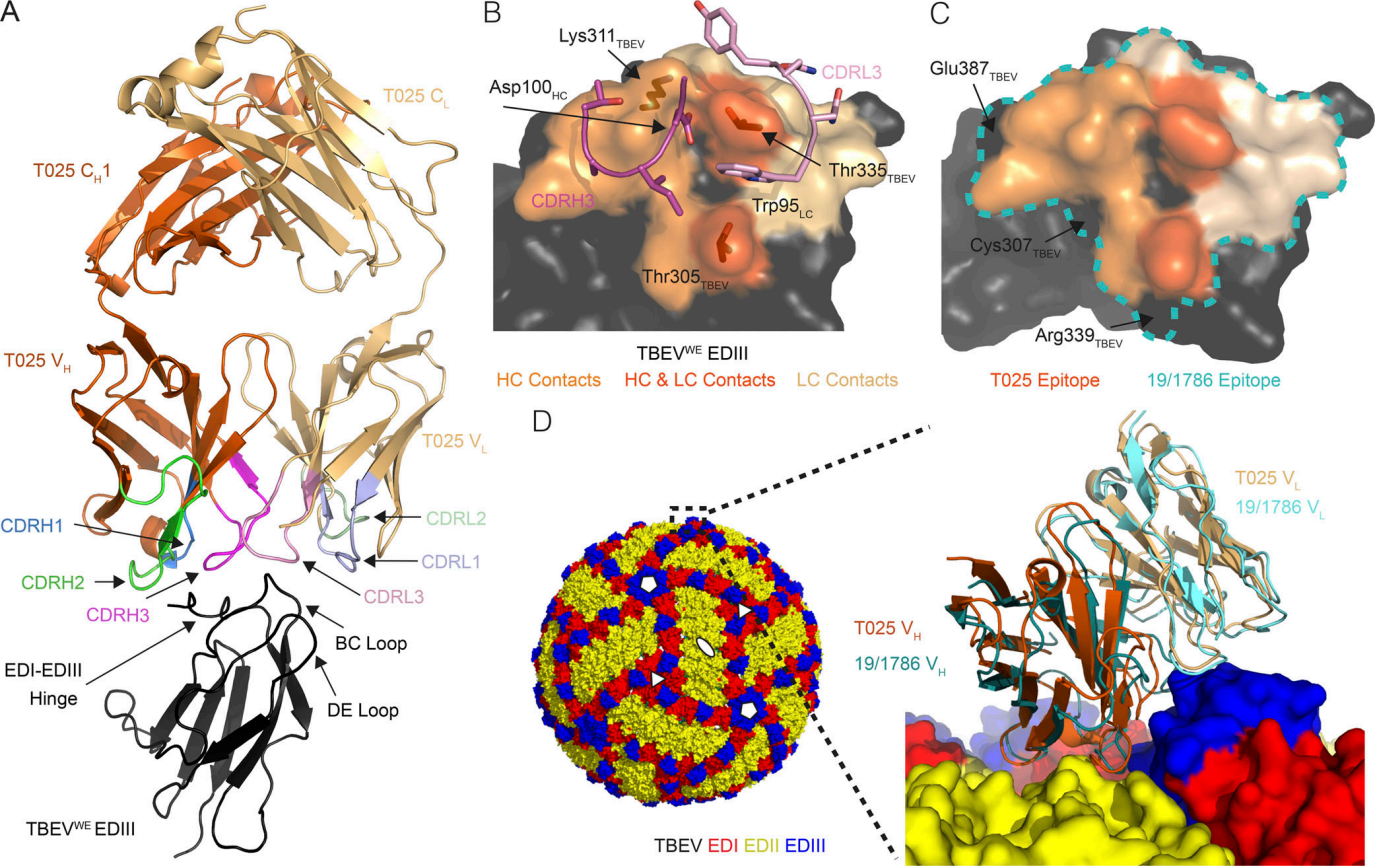
**T025 antibody recognizes a lateral ridge epitope on TBEV EDIII that is exposed on the mature virus structure.** **(A)** T025 recognition of the TBEV^WE^ EDIII. T025 interacts with the N-terminal region (EDI–EDIII hinge, the BC loop, and the DE loop) on TBEV^WE^ EDIII. **(B)** T025 epitope. TBEV^WE^ EDIII residues with an atom within 4 Å of a residue in the T025 Fab are highlighted on a surface representation of the EDIII antigen. CDRH3 and CDRL3 are shown as ribbon backbone with stick side chains. **(C)** T025 recognizes a similar epitope as the anti-TBEV mouse antibody 19/1786. The T025 epitope is shown in shades of orange; the 19/1786 epitope is outlined in a blue dashed line. Residues within the 19/1786 epitope, but not in the T025 epitope, are labeled. Epitopes are defined as residues that contain an atom within 4 Å of an atom in a residue on the antibody. **(D)** Surface representation of the cryo-EM structure of TBEV (PDB accession no. 5O6A) shown with fivefold, threefold, and twofold icosahedral symmetry operators at select vertices (left) with inset comparing binding poses of T025 and 19/1786 antibodies (right). Inset: Close-up of the indicated portion (dotted box) of the cryo-EM structure of the viral surface interacting with the 19/1786 V_H_V_L_ domains (PDB accession no. 5O6V) with the E domains labeled in red, yellow, and blue and the V_H_V_L_ domains in teal and cyan. The T025-TBEV^WE^ EDIII crystal structure was docked onto a virion EDIII adjacent to an icosahedral twofold symmetry axis after alignment of the EDIII domains (RMSD = 0.97 Å, 82 Ca atoms). The T025 V_H_V_L_ binds EDIII with a similar pose as the 19/1786 V_H_V_L_. Related to Fig. S5 and Table S6.

**Figure S5.**
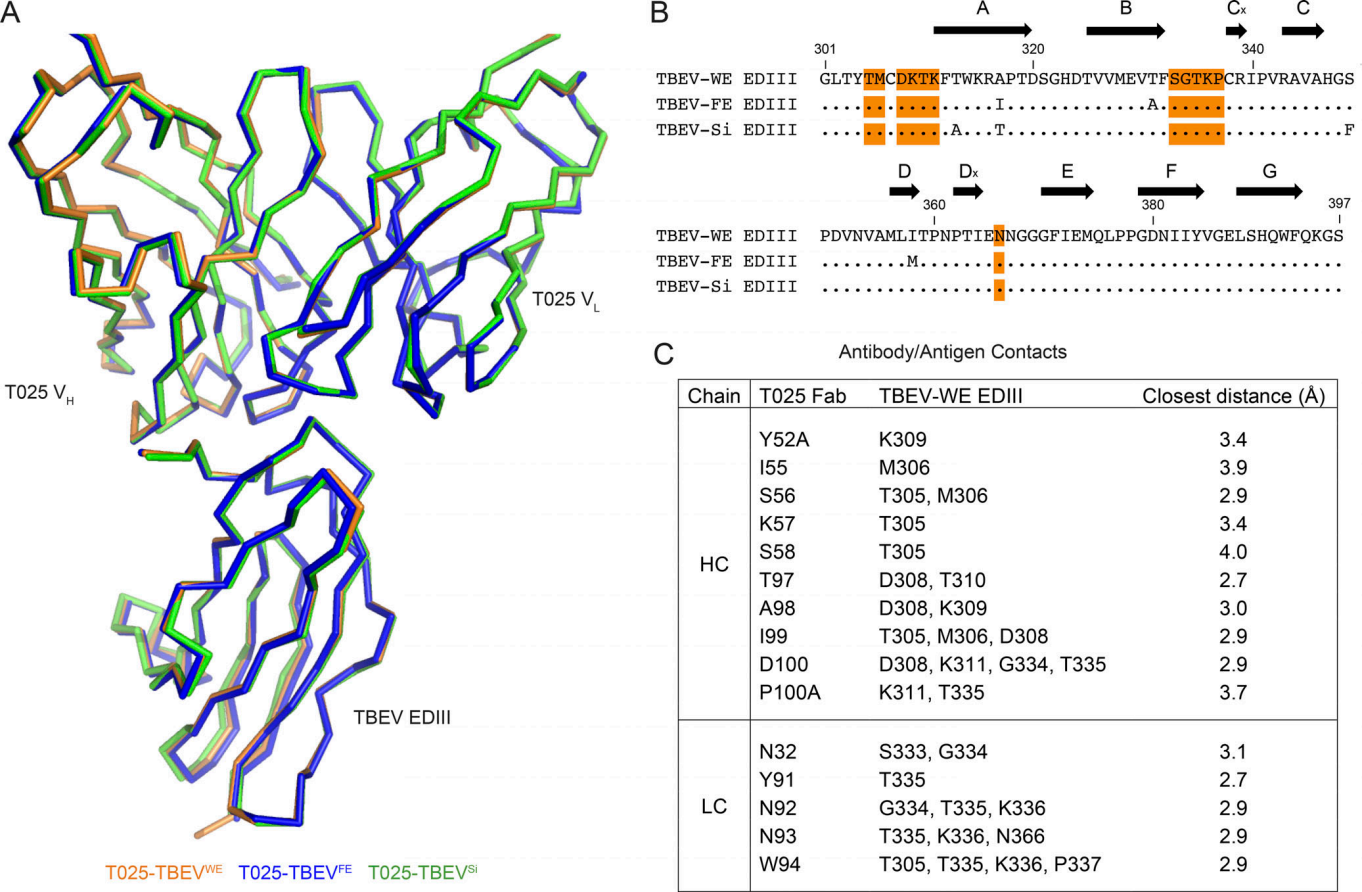
**T025 antibody recognizes three subtypes of TBEV EDIII.** **(A)** Overlay of crystal structures of T025 Fab bound to EDIII from three subtypes of TBEV. Structures were aligned on all Cα atoms in the Fab–EDIII complex and shown as orange (T025-TBEV^WE^ EDIII), blue (T025-TBEV^FE^ EDIII), or green (T025-TBEV^Si^ EDIII) ribbons. RMSDs for Fab-EDIII alignments are 0.54 Å (T025-TBEV^WE^ EDIII and T025-TBEV^FE^ EDIII; 518 Cα atoms) and 0.26 Å (T025-TBEV^WE^ EDIII and T025-TBEV^Si^ EDIII; 525 Cα atoms). The C_H_C_L_ domains of the Fabs were omitted for clarity. **(B)** Sequence alignment of EDIIIs from three strains of TBEV. Residues that contain an EDIII atom within 4 Å of an atom within a residue in T025 in each crystal structure are highlighted in orange. β-Strands are shown as arrows and defined according to Rey et al. (1995). **(C)** Contact residues between T025 Fab and TBEV^WE^ EDIII. Contacts were identified using AntibodyDatabase (West et al., 2013) and defined as residues in which any atom is within 4 Å of an atom from a residue on the interacting partner. Related to Fig. 4.

To gain insights into the mechanism of neutralization by human anti-TBEV antibodies, we solved crystal structures of the antigen-binding fragment (Fab) from the broad and potent antibody T025 (IGVH3–30/IGVK3–15) in complex with the EDIII domains of all three subtypes of TBEV (Fig. 4, Fig. S5, and Table S6). The structure of the T025 Fab–TBEV^WE^ EDIII complex revealed that the antibody binds the lateral ridge of EDIII in the proximity of the EDI–EDIII hinge region, making both heavy chain (HC) and light chain (LC) contacts to the EDI–EDIII hinge and the BC loop and light chain contacts to the DE loop of the EDIII (Fig. 4 A). The antibody contacts EDIII using CDRH2, CDRH3, CDRL1, and CDRL3, and buries 598 Å^2^ of surface area on the EDIII (333 Å^2^ by the V_H_ and 265 Å^2^ by the V_L_). T025 inserts Asp100_HC_ and Trp94_LC_ into a cleft in the EDIII, making a salt bridge (Asp100_HC_–Lys311_EDIII_) and hydrogen bonds to the EDIII (Fig. 4 B). Crystal structures of T025 Fab in complex with TBEV^FE^ EDIII and TBEV^Si^ EDIII were similar to the T025-TBEV^WE^ EDIII structure (root-mean-square deviations [RMSDs] = 0.54 Å for 518 Cα atoms and 0.26 Å for 525 Cα atoms, respectively), consistent with 100% sequence conservation between these three strains of virus in the EDIII epitope residues (Fig. S5).

We compared the T025 Fab–TBEV^WE^ structure to a 3.9 Å cryo-EM structure of a mouse monoclonal antibody (19/1786) bound to the TBEV virion (Füzik et al., 2018). T025 and 19/1786 are related by <65% amino acid sequence identity in the V_H_V_L_ and 47% in the CDRs, but structural alignment of the structures by the Cα atoms of the EDIIIs shows that the two antibodies recognize similar epitopes (Fig. 4 C) and adopt similar poses (Fig. 4 D). We can therefore use the lower-resolution cryo-EM structure to deduce details about how T025 binds to and neutralizes the virus. In addition to contacts with EDIII, 19/1786 interacts with either the EDI or EDII of a neighboring subunit (Füzik et al., 2018). This, taken together with the relatively low buried surface area on the EDIII by T025 (∼600 Å^2^ compared with a typical value of ∼1,100 Å^2^; Ramaraj et al., 2012), suggests that T025 also contacts neighboring domains on a native virion. It is also likely that, in common with recognition of virions by 19/1786, 120 of the 180 EDIIIs on the virion could be bound by T025.

### Antibody T025 prevents and treats infection in mice

**Figure 5.**
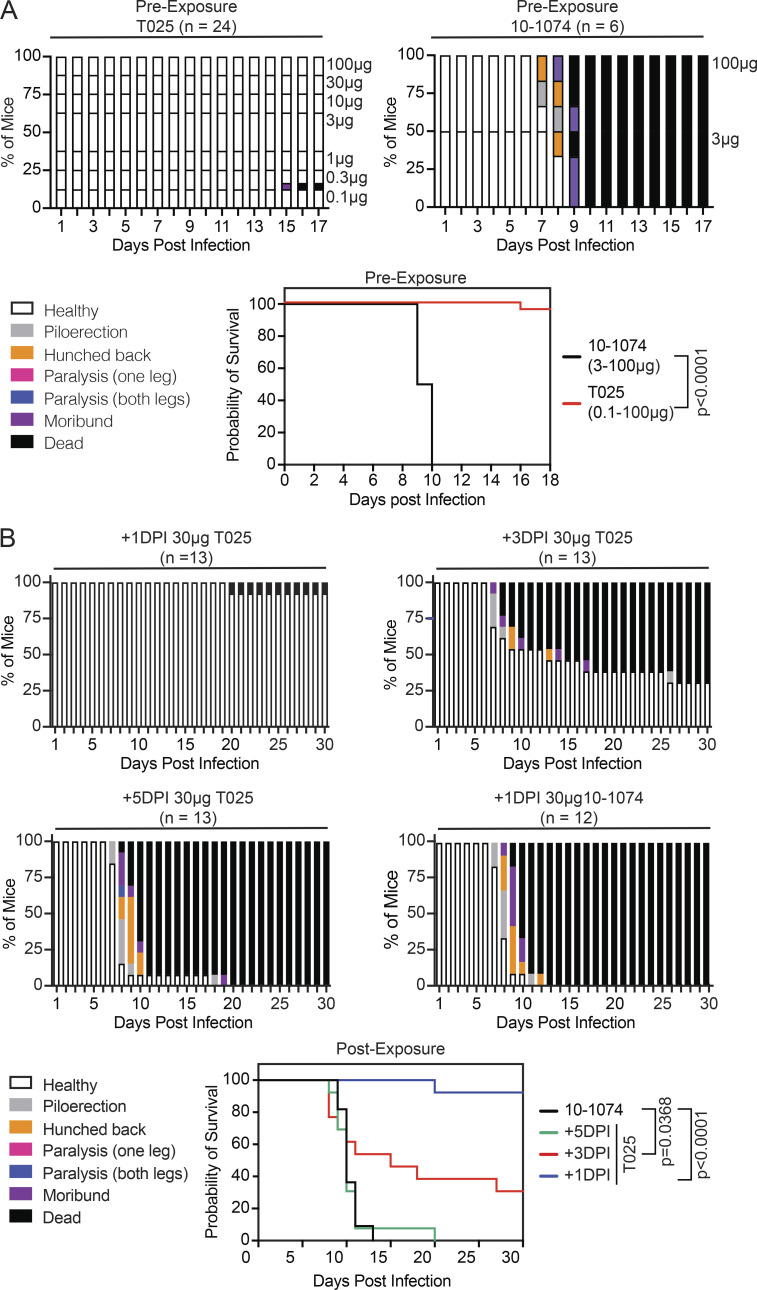
**Prevention and therapy with T025.** **(A)** T025 is efficacious in preexposure prophylaxis. Mice were treated with T025 or 10–1074 (isotype control) 24 h before infection with a lethal dose of TBEV-Hypr. Top: Histogram shows disease score over time. Antibody dose is indicated on the right. Two independent experiments were combined, with three mice per group. Bottom: Kaplan–Meier survival curve. The P value was calculated with the Mantel–Cox test (P < 0.0001). **(B)** T025 protects mice when administered after infection. Mice were treated with 30 µg T025 or control 10–1074 at 1, 3, or 5 d post infection (DPI). Three experiments were combined, with six or seven mice per group; P < 0.0001 for +1 DPI, P = 0.0368 for +3 DPI, and no significant difference for +5 DPI by Mantel–Cox test.

To determine whether anti-EDIII antibodies can protect against infection in vivo, we performed prophylaxis experiments in BALB/c mice. The mice received graded doses of T025 (100–0.1 µg per mouse) 24 h before challenge with 10^2^ PFU TBEV (a lethal dose). All mice treated with the isotype control antibody died by day 10 (*n* = 6). In contrast, even the lowest dose of T025 was protective; all but 1 of the 24 mice receiving the antibody survived (P < 0.0001; Fig. 5 A). To test T025’s potential for therapy, BALB/c mice were infected with 10^2^ PFU TBEV and then injected with 30 µg T025 or isotype control 1, 3, or 5 d later (Fig. 5 B). All 12 control mice succumbed to the infection by day 13. In contrast, 12 out of 13 mice treated with T025 on day 1 and 4 out of 13 mice treated on day 3 after infection survived. All mice treated with T025 5 d after infection failed to respond. Thus T025, a broadly neutralizing human anti-TBEV antibody, is efficacious in prevention and early treatment of TBEV infection in BALB/c mice.

## Discussion

Tick-borne flaviviruses can cause a fulminant encephalitis for which there is no effective therapy. This group of viruses are a growing public health concern in Europe, Asia, and North America. Among disease-causing tick-borne flaviviruses, TBEV is prevalent in Central Europe and Russia. Although there is a great deal of information on the polyclonal humoral immune response to TBEV (Albinsson et al., 2018; Holzmann, 2003; Matveeva et al., 1995; McAuley et al., 2017; Remoli et al., 2015), there is little or no understanding of the molecular nature of the neutralizing antibody response induced by natural infection or vaccination. We report on 776 antibodies obtained from memory B cells of six recovered and three vaccinated individuals, among which are several broad and potent neutralizers of tick-borne flaviviruses. The data provide insights into the human antibody response to TBEV and related pathogens, as well as mechanisms of antibody-induced neutralization. Finally, broad and potent neutralizing human monoclonal antibodies are highly effective for protection and therapy in vivo and have significant potential for clinical use.

Human neutralizing antibody responses to pathogens frequently converge on the same IGV genes. Examples include neutralizing antibodies to HIV-1, influenza, Zika, hepatitis B, and SARS-CoV-2 viruses (Robbiani et al., 2017; Robbiani et al., 2020; Scheid et al., 2011; Tiller et al., 2007; Wang et al., 2020; West et al., 2012). Antibodies to the EDIII of TBEV produced by different individuals show strong homology that, like SARS-CoV-2 antibodies, extends beyond IGV heavy and light chain gene pairing and includes the CDR3 regions.

Among the neutralizing antibodies to TBEV, VH1–69 and VH3–48 were recurrent in multiple donors. VH1–69 was paired with a variety of light chain genes to produce neutralizing antibodies that were found among vaccinees and recovered individuals. This group of antibodies varied broadly in neutralizing activity with IC_50_s ranging from 12–1,180 ng/ml (geometric mean, 186.2 ng/ml). VH1–69 antibodies are highly represented in the human repertoire and are also common among broadly neutralizing antibodies to influenza, hepatitis C, and HIV-1 viruses (Chen et al., 2019). Anti-TBEV VH3–48 antibodies differed from VH1–69 in that they were always paired with the same light chain, VK1–5. VH3–48 antibodies were also more potent than VH1–69, with IC_50_s ranging from 0.5 to 7.3 ng/ml (geometric mean, 2 ng/ml), and they were only found in convalescent individuals. The absence of this class of potent antibodies in the vaccinees examined is consistent with the lower levels of serum neutralizing potency in this group. Finally, VH3–48 antibodies are also potent neutralizers of several related tick-borne flaviviruses including KFDV, LGTV, LIV, and OHFV, with IC_50_s of 1–36 ng/ml.

Antibodies to a number of different flaviviruses, including dengue and Zika virus, can be protective if administered before and even after infection (Robbiani et al., 2017; Xu et al., 2017). In Russia and Kazakhstan, administration of TBEV hyperimmune plasma Ig is recommended for postexposure prophylaxis for individuals that present within 3 d of a tick bite (Pen’evskaia and Rudakov, 2010; Russian Ministry of Health, 2008). The efficacy of this intervention may vary from batch to batch of donor plasma Ig (Rabel et al., 2012; Ruzek et al., 2019), and its use was discontinued in some countries after a small number of adverse events and concerns about the possibility of antibody-dependent enhancement of disease (Arras et al., 1996; Kluger et al., 1995; Waldvogel et al., 1996). Mouse monoclonal antibodies can also protect against TBEV but have not been tested in the clinic (Baykov et al., 2014; Levanov et al., 2010; Matveev et al., 2020). Our experiments extend previous work by uncovering human monoclonal antibodies that prevent infection in mice even when administered at doses as low as ∼0.005 mg/kg. Notably, these antibodies also suppress disease in mice, even when administered 3 d after infection at a dose of ∼1.5 mg/kg.

Antibodies against EDIII are often among the most potent raised during flavivirus infection (Beasley and Barrett, 2002; Beltramello et al., 2010; Crill and Roehrig, 2001; Screaton et al., 2015; Sun et al., 2017). The broad and potent anti-TBEV antibody T025 recognizes a lateral ridge epitope on EDIII that is also targeted by antibodies raised against other flaviviruses (Edeling et al., 2014; Füzik et al., 2018; Nybakken et al., 2005; Renner et al., 2018; Robbiani et al., 2017; Zhao et al., 2020). Several human antibodies against the lateral ridge of EDIII have been previously characterized, including Z021 isolated from a convalescent Zika donor, which recognizes a similar epitope and has an angle of approach similar to T025 (Keeffe et al., 2018). These results suggest a common mechanism for potent neutralization of flaviviruses. Targeting this epitope likely interferes with the structural rearrangement necessary for fusion, preventing infection (Füzik et al., 2018; Nybakken et al., 2005; Renner et al., 2018; Thompson et al., 2009). Although anti-EDIII antibodies can be potently neutralizing, antibodies against the EDIII in humans constitute only a small fraction of the immune response (Beltramello et al., 2010; Lai et al., 2008; Wahala et al., 2009), suggesting that a targeted vaccine strategy focusing on EDIII could be beneficial.

While most antibodies to flavivirus EDIII are thought to be virus specific (Crill and Roehrig, 2001; Pierson et al., 2008; Roehrig, 2003; Stettler et al., 2016), T025 is a potent neutralizer of not only TBEV but also several other tick-borne flaviviruses. The pairwise sequence identity of the EDIII epitope of TBEV to predicted epitopes in LGTV, LIV, and OHMV EDIIIs is high (differences of 1–2 amino acids), and the residues that differ have similar biochemical properties (e.g., threonine to serine). The exception is KFDV, which is sensitive to T025 but differs in sequence to TBEV in the T025 epitope on the EDIII. Contacts made outside of the EDIII may also contribute to the sensitivity of KDFV to T025. Powassan virus contains an amino acid insertion in the BC loop of the EDIII compared with the TBEV EDIII, which likely prevents binding of T025 and therefore is insensitive to T025 neutralization.

Available TBEV vaccines were developed over 30 yr ago and consist of inactivated virus grown on chick embryo cells. Vaccination is TBEV specific, requires priming and two boosts, and results in 90–100% seroconversion depending on the vaccine used (Loew-Baselli et al., 2009; Maikova et al., 2019; Vorovitch et al., 2019). Additional boosts are recommended every 3–5 yr for the lifetime of the individual. The existence of broad and potent VH3–48 antibodies suggests that next-generation vaccines specifically designed to target the epitope recognized by these antibodies might be universally effective against TBEV, KFDV, LGTV, LIV, and OHFV. Finally, potent human antibodies with broad activity against tick-borne flaviviruses have significant potential for clinical use in individuals who are at high risk and do not respond to the vaccine, as well as for therapy in the early stages of infection.

## Materials and methods

### Human subjects and clinical information

Samples of peripheral blood were obtained upon consent from individuals previously hospitalized with confirmed TBEV infection or individuals previously vaccinated against TBEV in České Budějovice, Czech Republic, under protocols approved by the ethical committees of the Hospital in České Budějovice (approval no. 103/19), the Biology Center of the Czech Academy of Sciences (approval no. 1/2018), and The Rockefeller University (IRB DRO-0984). Clinical data were obtained at the treating hospital, and severity of disease was evaluated according to the following scale: mild, flu-like symptoms with meningeal irritation defined as meningitis, characterized by fever, fatigue, nausea, headache, back pain, arthralgia/myalgia and neck or back stiffness; moderate, previous symptoms together with tremor, vertigo, somnolence, and photophobia defined as meningoencephalitis; severe, prolonged neurological consequences including ataxia, titubation, altered mental status, memory loss, quantitative disturbance of consciousness, and palsy revealed as encephalitis, encephalomyelitis, or encephalomyeloradiculitis (Bogovic and Strle, 2015; Ruzek et al., 2019).

### Blood samples processing and storage

Peripheral blood mononuclear cells (PBMCs) were obtained by gradient centrifugation using Ficoll and stored in liquid nitrogen in freezing media (90% FCS and 10% DMSO). Prior to experiments, aliquots of sera (from infected, vaccinated, and random blood bank donors) were heat-inactivated at 56°C for 1 h and then stored at 4°C.

### Protein expression and purification

EDIII antigens were expressed in *Escherichia coli* and purified from inclusion bodies as previously reported (Robbiani et al., 2017; Sapparapu et al., 2016). Expression vectors containing codon-optimized sequences encoding residues 299–397 for TBEV strain Neudoerfl (TBEV^WE^; NC_001672.1) or 301–397 for strains Sofjin (TBEV^FE^; UniProtKB accession no. P07720) and Vasilchenko (TBEV^Si^; AF069066) were used to produce untagged EDIII proteins or EDIII proteins containing a C-terminal 6XHis-Avitag. Constructs encoding untagged EDIIIs of other tick-borne flaviviruses were constructed similarly (POWV strain LB, GenBank accession nos. L06436.1; POWV isolate DTV, HM440561.1; KFDV strain W-377, JF416960.1; LGTV strain TP21-636, NC_003690.1; LIV isolate LI3/1, KP144331.1; OHFV strain Bogoluvovska, NC_005062). Expression plasmids were transformed into BL21(DE3) *E. coli* and induced with 1 mM isopropyl β-D-1-thiogalactopyranoside at 37°C for 4 h. The cells were lysed and the insoluble fraction containing inclusion bodies was solubilized and refolded in 400 mM L-arginine, 100 mM Tris-base, pH 8.0, 2 mM EDTA, 0.2 mM phenyl-methylsulfonyl fluoride, 5 mM reduced and 0.5 mM oxidized glutathione, and 10% glycerol at 4°C. Refolded protein was purified by size exclusion chromatography (Cytiva; Superdex 75) in 20 mM Tris, pH 8.0, 150 mM NaCl, and 0.02% NaN_3_. EDIIIs were concentrated to 10–20 mg/ml.

T025 Fabs for structural studies were produced and purified as described in previous studies (Keeffe et al., 2018; Robbiani et al., 2017; Robbiani et al., 2020; Wang et al., 2020). Briefly, Fabs containing a 6XHis purification tag at the C terminus of the HC were expressed by transiently transfecting Expi293 cells (Life Technologies) with appropriate heavy and light chain plasmids. His-tagged Fabs were purified from expression supernatants using Ni-nitrilotriacetic acid affinity chromatography (Cytiva) followed by size exclusion chromatography (Cytiva; Superdex 200) in 20 mM Tris, pH 8.0, 150 mM NaCl, and 0.02% NaN_3_. Fabs were concentrated to ∼15 mg/ml.

### Sequence analysis

Antibody sequences were analyzed as described previously (Robbiani et al., 2020); briefly, sequences were trimmed and annotated using Igblastn v.1.14.0 (Ye et al., 2013) and Change-O toolkit v.0.4.5 (Gupta et al., 2015). Sequences from the same cell were paired and assigned clonotypes based on V and J genes using in-house R and Perl scripts (available on GitHub; https://github.com/stratust/igpipeline). Nucleotide somatic hypermutation and CDR3 length were also analyzed using in-house R and Perl scripts, as described previously (Robbiani et al., 2020); hypermutation analysis was based on the closest germlines in Igblastn. Hydrophobicity GRAVY (grand average of hydropathy) scores were calculated using Guy H.R. Hydrophobicity scale (Guy, 1985; Kyte and Doolittle, 1982) and R package Peptides (https://journal.r-project.org/archive/2015/RJ-2015-001/RJ-2015-001.pdf), based on 776 IGH CDR3 sequences from this study and 22,654,256 IGH CDR3 sequences from public databases of memory B cell receptor sequences (DeWitt et al., 2016). Distribution was determined using the Shapiro–Wilk test with all CDR3 sequence GRAVY scores from this study and 5,000 randomly selected GRAVY scores from the public database. The Wilcoxon nonparametric test was used to test for significant difference in hydrophobicity.

Frequency distributions of V genes in anti-TBEV antibodies from six infected donors were compared with Sequence Read Archive accession no. SRP010970 (https://trace.ncbi.nlm.nih.gov/Traces/sra/?study=SRP010970; Rubelt et al., 2012). V gene assignments were based on the above-described analysis, and frequencies were calculated for six infected donors using sequences with unique CDR3s. Statistical significance was determined using two-tailed *t* tests with unequal variances. Sequence logos were generated from left-aligned CDR3 sequences from each antibody set using WebLogo (Crooks et al., 2004).

### Protein biotinylation

Avi-tagged TBEV^FE^ EDIII was biotinylated using the Biotin-Protein Ligase BIRA kit according to the manufacturer’s instructions (Avidity) and conjugated to streptavidin-PE (BD Biosciences; 554061) and streptavidin-Alexa Fluor 647 (BioLegend; 405237). Ovalbumin (Sigma-Aldrich; A5503-1G) was biotinylated using the EZ Sulfo-NHS-LC-Biotinylation kit according to the manufacturer’s instructions (Thermo Fisher Scientific; A39257) and conjugated to streptavidin BV711 (BD Biosciences; 563262). Biotinylation was confirmed by ELISA before use in flow cytometry.

### Single-cell sorting

PBMCs from sample 111 were enriched for B cells via positive selection using CD19 microbeads (Miltenyi Biotec; 130–050-301). PBMCs from all other donors were enriched for B cells by negative selection (Miltenyi Biotec; 130–101-638). All selection protocols were performed according to the manufacturer’s instructions. Enriched B cells were incubated for 30 min on ice in FACS buffer (1× PBS, 2% calf serum, 1 mM EDTA) with fluorophore-labeled EDIII and ovalbumin, and in the presence of anti-human antibodies anti-CD3-APC-eFluro 780 (Invitrogen; 47–0037-41), anti-CD8-APC-eFluro 780 (Invitrogen; 47–0086-42), anti-CD14-APC-eFluro 780 (Invitrogen; 47–0149-42), anti-CD16-APC-eFluro 780 (Invitrogen; 47–0168-41), anti-CD20-PECy7 (BD Biosciences; 335793), and Zombie NIR (BioLegend; 423105). Single CD3^−^CD8^−^CD14^−^CD16^−^ZombieNIR^−^CD20^+^Ova^−^EDIII-PE^+^EDIII-AF647^+^ B cells were sorted using a FACS Aria III (Becton Dickinson) into individual wells of 96-well plates. Each well contained 4 µl of a lysis buffer comprising 0.5× PBS, 10 mM DTT, and 3,000 U/ml RNasin Ribonuclease Inhibitors (Promega; N2615). Sorted cells were snap-frozen on dry ice and then stored at −80°C. Antibody sequences are derived from memory B cells because they originate from small CD20^+^ cells, and the antibody genes were PCR amplified using IgG-specific primers.

### Antibody sequencing, cloning, and expression

RNA from single cells was reverse transcribed using SuperScript III Reverse transcription (Invitrogen; 18080–044). The resulting cDNA was stored at −20°C until amplification of the variable Ig heavy (IGH), Ig light (IGL), and Ig kappa (IGK) genes by nested PCR followed by Sanger sequencing. Amplicons from the first PCR reaction were used as template for nested PCR-amplification followed by sequence- and ligation-independent cloning into antibody expression vectors as previously described (Robbiani et al., 2020). Recombinant monoclonal antibodies were produced and purified as previously detailed (Klein et al., 2014).

### Plasmids for the production of RVP

A West Nile virus subgenomic replicon-expressing plasmid encoding Renilla luciferase (pWNVII-Rep-REN-IB) and a ZIKV CprME expression plasmid had previously been obtained from Ted Pierson (National Institutes of Health, Bethesda, MD; Pierson et al., 2006; Robbiani et al., 2017). The ZIKV CprME expression plasmid was manipulated by restriction enzyme digestion and ligation to express the CprME of other flaviviruses as follows.

#### TBEV

Synthetic DNA with CprME coding sequence (flanked at the 5′ by the polylinker and Kozak sequence 5′-GGA​ATT​CGC​GGC​CGC​CTC​AGG-3′ and at the 3′ by the stop codons and polylinker 5′-TAA​TAG​TTA​ATT​AAC​TCG​AGC​CGC​GG-3′; “CprME flanked”) corresponding to TBEV, Western European subtype strain Neudoerfl (GenBank accession no. NC_001672), was amplified with primers DFRp1532 (5′-GGA​ATT​CGC​GGC​CGC​CTC​AGG-3′) and DFRp1533 (5′-GCG​GCT​CGA​GTT​AAT​TAA-3′) before cloning at the NotI and PacI sites of plasmid pPOWV-LB-CprME (see below), resulting in pTBEV-WE-CprME.

#### POWV-LB

Synthetic DNA containing the CprME sequence (underlined) of POWV-LB strain (GenBank accession no. L06436.1 with six synonymous changes (in lowercase and bold) to reduce complexity; 5′-CTA​CTT​GGC​AGT​ACA​TCT​ACG​TAT​TAG​TCA​TCG​CTA​TTA​CCA​TGG​TGA​TGC​GGT​TTT​GGC​AGT​ACA​TCA​ATG​GGC​GTG​GAT​AGC​GGT​TTG​ACT​CAC​GGG​GAT​TTC​CAA​GTC​TCC​ACC​CCA​TTG​ACG​TCA​ATG​GGA​GTT​TGT​TTT​GGC​ACC​AAA​ATC​AAC​GGG​ACT​TTC​CAA​AAT​GTC​GTA​ACA​ACT​CCG​CCC​CAT​TGA​CGC​AAA​TGG​GCG​GTA​GGC​GTG​TAC​GGT​GGG​AGG​TCT​ATA​TAA​GCA​GAG​CTC​TCT​GGC​TAA​CTA​GAG​AAC​C**C**ACT​GCT​TAC​TGG​CTT​ATC​GAA​ATT​AAT​ACG​ACT​CAC​TAT​AGG​GAG​ACC​CAA​GCT​GGC​TAG​TTA​AGC​TAT​CAA​CAA​GGA​ATT​CGC​GGC​CGC​CAG​GCTATG​ATG​ACC​ACT​TCT​AAA​GGA​AAG​GGG​GGC​GGT​CCC​CCT​AGG​CGC​AAG​CTT​AAA​GTG​ACC​GCA​AAT​AAG​TCG​CGA​CCA​GCA​ACG​AGC​CCA​ATG​CCA​AAG​GGC​TTC​GTG​CTG​TCG​CGC​ATG​CTG​GGG​ATT​CTT​TGG​CAC​GCC​GTG​ACA​GGC​ACG​GCC​AGA​CCC​CCA​GTG​CTG​AAA​ATG​TTC​TGG​AAA​ACG​GTA​CCA​CTG​CGC​CAG​GCG​GAG​GCT​GTT​CTG​AAG​AAG​ATA​AAG​AGA​GTT​ATC​GGG​AAC​TTG​ATG​CAG​AGC​CTT​CAC​ATG​AGA​GGG​CGT​CGC​AGG​TCA​GGT​GTG​GAC​TGG​ACT​TGG​ATT​TTT​TTG​ACG​ATG​GCG​TTG​ATG​ACC​ATG​GCC​ATG​GCA​ACC​ACC​ATC​CAC​CGG​GAC​AGG​GAA​GGA​TAC​ATG​GTT​ATG​CGG​GCC​AGT​GGA​AGG​GAC​GCT​GCA​AGC​CAG​GTC​AGG​GTA​CAA​AAC​GGA​ACG​TGC​GTC​ATC​CTG​GCA​ACA​GAC​ATG​GGA​GAG​TGG​TGT​GAA​GAT​TCA​ATC​ACC​TAC​TCT​TGC​GTC​ACG​ATT​GAC​CAG​GAG​GAA​GAA​CCC​GTT​GAC​GTG​GAC​TGC​TTC​TGC​CGA​GGT​GTT​GAT​AGG​GTT​AAG​TTA​GAG​TAT​GGA​CGC​TGT​GGA​AGG​CAA​GCT​GGA​TCT​AGG​GGG​AAA​AGG​TCT​GTG​GTC​ATT​CCA​ACA​CAT​GCA​CAA​AAA​GAC​ATG​GTC​GGG​CGA​GGT​CAT​GCA​TGG​CTT​AAA​GGT​GAC​AAT​ATT​CGA​GAT​CAT​GTC​ACC​CGA​GTC​GAG​GGC​TGG​ATG​TGG​AAG​AAC​AAG​CTT​CTA​ACT​GCC​GCC​ATT​GTG​GCC​TTG​GCT​TGG​CTC​ATG​GTT​GAT​AGT​TGG​ATG​GCC​AGA​GTG​ACT​GTC​ATC​CTC​TTG​GCG​TTG​AGT​CTA​GGG​CCA​GTG​TAC​GCC​ACG​AGG​TGC​ACG​CAT​CTT​GAG​AAC​AGA​GAT​TTT​GTG​ACA​GGA​ACT​CAA​GGG​ACC​ACC​AGA​GTG​TCC​CTA​GTT​TTG​GAA​CTT​GGA​GGC​TGC​GTG​ACC​ATC​ACA​GCT​GAG​GGC​AAG​CCA​TCC​ATT​GAT​GTA​TGG​CTC​GAA​GAC​ATT​TTT​CAG​GAA​AGC​CCG​GCT​GAA​ACC​AGA​GAA​TAC​TGC​CTG​CAC​GCC​AAA​TTG​ACC​AAC​ACA​AAA​GTG​GAG​GCT​CGC​TGT​CCA​ACC​ACT​GGA​CCG​GCG​ACA​CTT​CCG​GAG​GAG​CAT​CAG​GCT​AAT​ATG​GTG​TGC​AAG​AGA​GAC​CAA​AGC​GAC​CGT​GGA​TGG​GGA​AAC​CAC​TG**t**GG**a**TT**c**TT**c**GGG​AAG​GGC​AGT​ATA​GTG​GCT​TGT​GCA​AAG​TTT​GAA​TGC​GAG​GAA​GCA​AAA​AAA​GCT​GTG​GGC​CAC​GTC​TAT​GAC​TCC​ACA​AAG​ATC​ACG​TAT​GTT​GTC​AAG​GTT​GAG​CCC​CAC​ACA​GGG​GAT​TAC​TTG​GCT​GCA​AAT​GAG​ACC​AAT​TCA​AAC​AGG​AAA​TCA​GCA​CAG​TTT​ACG​GTG​GCA​TCC​GAG​AAA​GTG​ATC​CTG​CGG​CTC​GGC​GAC​TAT​GGA​GAT​GTG​TCG​CTG​ACG​TGT​AAA​GTG​GCA​AGT​GGG​ATT​GAT​GTC​GCC​CAA​ACT​GTG​GTG​ATG​TCA​CTC​GAC​AGC​AGC​AAG​GAC​CAC​CTG​CCT​TCT​GCA​TGG​CAA​GTG​CAC​CGT​GAC​TGG​TTT​GAG​GAC​TTG​GCG​CTG​CCC​TGG​AAA​CAC​AAG​GAC​AAC​CAA​GAT​TGG​AAC​AGT​GTG​GAG​AAA​CTT​GTG​GAA​TTT​GGA​CCA​CCA​CAT​GCT​GTG​AAA​ATG​GAT​GTT​TTC​AAT​CTG​GGG​GAC​CAG​ACG​GCT​GTG​CTG​CTC​AAA​TCA​CTG​GCA​GGA​GTT​CCG​CTG​GCC​AGT​GTG​GAG​GGC​CAG​AAA​TAC​CAC​CTG​AAA​AGC​GGC​CAT​GTT​ACT​TGT​GAT​GTG​GGA​CTG​GAA​AAG​CTG​AAA​CTG​AAA​GGC​ACA​ACC​TAC​TCC​ATG​TGT​GAC​AAA​GCA​AAG​TTC​AAA​TGG​AAG​AGA​GTT​CCT​GTG​GAC​AGC​GGC​CAT​GAC​ACA​GTA​GTC​ATG​GAG​GTA​TCA​TAC​ACA​GGA​AGC​GAC​AAG​CCA​TGT​CGG​ATC​CCG​GTG​CGG​GCT​GTG​GCA​CAT​GGT​GTC​CCA​GCG​GTT​AAT​GTA​GCC​ATG​CTC​ATA​ACC​CCC​AAT​CCA​ACC​ATT​GAA​ACA​AAT​GGT​GGC​GGA​TTC​ATA​GAA​ATG​CAG​CTG​CCA​CCA​GGG​GAT​AAC​ATC​ATC​TAT​GTG​GGA​GAC​CTT​AGC​CAG​CAG​TGG​TTT​CAG​AAA​GGC​AGT​ACC​ATT​GGT​AGA​ATG​TTT​GAA​AAA​ACC​CGC​AGG​GGA​TTG​GAA​AGG​CTC​TCT​GTG​GTT​GGA​GAA​CAT​GCA​TGG​GAC​TTT​GGC​TCA​GTA​GGC​GGG​GTA​CTG​TCT​TCT​GTG​GGG​AAG​GCA​ATC​CAC​ACG​GTG​CTG​GGG​GGA​GCT​TTC​AAC​ACC​CTT​TTT​GG**t**GG**t**GTT​GGA​TTC​ATC​CCT​AAG​ATG​CTG​CTG​GGG​GTT​GCT​CTG​GTC​TGG​TTG​GGA​CTA​AAT​GCC​AGG​AAT​CCA​ACG​ATG​TCC​ATG​ACG​TTT​CTT​GCT​GTG​GGG​GCT​TTG​ACA​CTG​ATG​ATG​ACA​ATG​GGA​GTT​GGG​GCATAA​TAG​TTA​ATT​AAC​TCG​AGC​CGC​GGT​TCG​AAG​GTA​AGC​CT-3′) was PCR amplified with primers DFRp1511 (5′-ATC​TAC​GTA​TTA​GTC​ATC​GCT​ATT​A-3′) and DFRp1514 (5′-ACC​GCG​GCT​CGA​GTT​AAT​TAA-3′) and cloned at the Eco105I and SacII sites of plasmid pZIKV-HPF-CprME (Pierson et al., 2006; Robbiani et al., 2017), resulting in pPOWV-LB-CprME.

#### POWV-DTV

A three-piece assembly PCR strategy was used. DNA upstream of the CMV promoter in pZIKV-HFP-CprME to just downstream of the beginning of the C-encoding region was PCR amplified with primers RU-O-24611 (5′-CTT​GAC​CGA​CAA​TTG​CAT​GAA​G-3′) and RU-O-26690 (5′-**CTT​TCC​TTT​AGA​AGT​AGT​CAC​CAT**AGC​CTG​CTT​TTT​TGT​ACA​AAC-3′), resulting in a fragment fusing the CMV promoter with POWV-DTV C-encoding sequences (bolded in primer RU-O-26690). Using as template DTVp1 (Kenney et al., 2018), kindly provided by Aaron Brault (Centers for Disease Control and Prevention, Fort Collins, CO) and based on the Spooner strain of DTV, a fragment overlapping with the CMV promoter–DTV C fusion to the region just downstream of a SacII site within DTV genome was generated by PCR using oligos RU-O-26689 (5′-GTT​TGT​ACA​AAA​AAG​CAG​GCT**ATG​GTG​ACT​ACT​TCT​AAA​GGA​AAG**-3′) and RU-O-26711 (5′-GTT​TCC​CCA​TCC**T**C**TA**TCG​CTC​TG-3′), with bolded nucleotides indicating synonymous mutations introduced to ablate the SacII site. DNA was amplified using DTVp1 as template and oligos RU-O-26710 (5′-CAG​AGC​GA**TA**G**A**GGA​TGG​GGA​AAC-3′; bolded nucleotides indicate synonymous mutations) and RU-O-26688 (5′-TTC​GAA​CCG​CGG​CTG​GGT​CCT​ATT​A**TGC​TCC​GAC​TCC​CAT​TGT​CAT​CAT​C**-3′) to generate a fragment overlapping the killed SacII site to the end of the E protein coding region followed by a SacII site. The three DNA fragments were annealed, extended and then PCR amplified using primers RU-O-24611 and RU-O-26688. The resulting DNA fragment was digested with SnaBI and SacII and cloned into similarly digested pZIKV-HPF-CprME to generate pPOWV-DTV-CprME.

#### KFDV

Synthetic DNA with the CprME-flanked sequence of KFDV, strain W-377 (GenBank accession no. JF416960.1), was amplified with primers DFRp1532 and DFRp1533 before cloning at the NotI and PacI sites of plasmid pPOWV-LB-CprME (see above), resulting in pKFDV-W-377-CprME.

#### LGTV

The CprME of LGTV, isolate TP21-636, was amplified from a plasmid kindly provided by Dr. Sonja Best (Rocky Mountain Laboratories of National Institutes of Health/National Institute of Allergy and Infectious Diseases, Hamilton, MT) with primers DFRp1563 (5′-GGA​ATT​CGC​GGC​CGC​CTC​AGG​ATG​GCC​GGG​AAG​GCC​GTT​CTA-3′) and DFRp1566 (5′-CCG​CGG​CTC​GAG​TTA​ATT​AAC​TAT​TAG​GCT​CCA​ACC​CCC​AGA​GTC​AT-3′) before cloning at the NotI and PacI sites of plasmid pPOWV-LB-CprME, resulting in pLGTV-TP21-636-CprME. There are two nucleotide mutations from GenBank accession no. NC_003690 (A590G and A1893C).

#### LIV

Synthetic DNA with the CprME-flanked sequence of LIV, isolate LI3/1 (GenBank accession no. KP144331), was amplified with primers DFRp1532 and DFRp1533 before cloning at the NotI and PacI sites of plasmid pPOWV-LB-CprME, resulting in pLIV-LI3/1-CprME.

#### OHFV

Synthetic DNA with the CprME-flanked sequence of OHFV, strain Bogoluvovska (GenBank accession no. NC_005062), was amplified with primers DFRp1532 and DFRp1533 before cloning at the NotI and PacI sites of plasmid pPOWV-LB-CprME, resulting in pOHFV-CprME.

To confirm the absence of PCR-induced errors, all PCR-derived regions were sequenced in the final plasmids.

### RVP production

RVPs were produced by cotransfecting 1 µg pWNVII-Rep-REN-IB plasmid with 3 µg of the flavivirus CprME plasmid of choice into the permissive cell line Lenti-X 293T using Lipofectamine 2000 (Invitrogen; 1166803) according to the manufacturer’s instructions. Cells were seeded 24 h previously at 10^6^ cells/well in collagen-coated 6-well plates. Following transfection and 6 h incubation at 37°C, excess DNA–lipid complexes were removed by aspiration and the media replaced with DMEM (Gibco) containing 20 mM Hepes and 10% FBS. For the next 72 h, in 24-h intervals, RVP-containing supernatants were harvested and filtered through a 0.45-μm filter and frozen at −80°C, and media were replaced with DMEM containing 20 mM Hepes and 10% FBS. Frozen RVPs were later thawed and titrated on Huh-7.5 cells to determine the dilution of RVPs at which cells express 10^6^ relative light units in the absence of sera or antibody.

### RVP neutralization assays

96-well plates were seeded with 7,500 Huh-7.5 cells/well in 50 µl DMEM (Gibco) supplemented with 10% FBS and 1% nonessential amino acids. After 24 h, 100 µl diluted RVPs were combined with 100 µl diluted sera or antibody, incubated for 1 h at 37°C, and then 50 µl of the mix were added in triplicate to the plated cells. RVPs are diluted appropriately in BA-1 diluent (Medium 199 [Lonza] supplemented with 1% BSA and 100 units/ml penicillin/streptomycin) to achieve the desired relative light unit expression. After an additional 24 h of incubation at 37°C, media was aspirated off the cells, replaced with 35 µl lysis buffer (Promega; E2810), and the plates were frozen at −80°C. 15 µl of the subsequently thawed lysis buffer was used for Renilla luciferase expression measurement using the Renilla Luciferase Assay System (Promega; E2810). Sera were either diluted to 1:600,000 final concentration for TBEV RVP neutralization screening or serially diluted to generate curves. Recombinant monoclonal antibodies were used at 10 µg/ml final concentration and serially diluted 1:3 for neutralization assays. NT_50_ and IC_50_ were determined by nonlinear regression analysis using Prism software (GraphPad). In the cross-neutralization screening against the panel of flavivirus RVPs, recombinant antibodies were assayed at 1 µg/ml final concentration using the protocol described above, and the results were compared with no antibody control.

### ELISA assays

Binding of serum IgG or recombinant IgG antibodies to EDIII proteins was measured by standard ELISA. High-binding 96-well plates (Costar; 07–200-721) were coated overnight with 250 ng of the EDIII protein in PBS per well at room temperature; plates were then blocked with 0.1 mM EDTA, 0.05% Tween, and 2% BSA in PBS for 2 h at room temperature. Samples were diluted in PBS-T, added to plates, and incubated for an additional 1 h at room temperature. Secondary goat anti-human-IgG Fab′_2_ fragments conjugated to HRP (Jackson ImmunoResearch; 109–036-088) were diluted 1:5,000 in PBS-T, added to the plates, and incubated again for 1 h at room temperature. Between each step, the plates were washed with PBS-T four times. Plates were finally developed using TMB substrate (Thermo Fisher Scientific; 34021); the reaction was stopped using 1 M sulfuric acid and the plates read at 450 nm. Sera were screened for binding at 1:500 dilution. Recombinant monoclonal antibodies were diluted to 10 µg/ml and serially diluted 1:3; the half effective concentration (EC_50_) was determined by nonlinear regression analysis using Prism 8 (GraphPad). For cross-binding assays, recombinant antibodies were assayed at 1 µg/ml according to the protocol described above using the panel of flavivirus EDIII proteins. The anti-HIV monoclonal antibody 10–1074 was used as isotype control (Mouquet et al., 2012). Antibodies with optical density >2.5 times isotype control signal were considered cross-reactive. The TBEV clinical tests (Table S1) were conducted using the EIA TBE Virus IgG (TBG096) and EIA TBE Virus IgM (TBM096) kits from TestLine Clinical Diagnostics.

### Viruses and cells

The low-passage TBEV strain Hypr was provided by the Collection of Arboviruses, Institute of Parasitology, Biology Centre of the Czech Academy of Sciences, Ceske Budejovice, Czech Republic (http://www.arboviruscollection.cz/index.php?lang=en). The virus was originally isolated from the blood of a diseased 10-yr-old child in Brno, Czech Republic (formerly Czechoslovakia), in 1953. Prior to the use in in vitro and in vivo experiments, the virus was propagated in suckling mouse brains and/or BHK-21 cells.

PS (porcine kidney stable) cells (Kozuch and Mayer, 1975) were cultured at 37°C in Leibovitz (L-15) medium supplemented with 3% FBS, 100 U/ml penicillin, 100 µg/ml streptomycin, and 1% L-glutamine (Sigma-Aldrich).

### Plaque assay

To determine virus titer, plaque assays were performed as previously described (De Madrid and Porterfield, 1969), with slight modifications (Pokorna Formanova et al., 2019). Briefly, 10-fold dilutions of virus plus a suspension of PS cells (1.3 × 10^5^ cells per well) were added to 24-well tissue culture plates. After 4 h of incubation at 37°C with 0.5% CO_2_, each well was overlaid with carboxymethylcellulose (1.5% in L-15 medium). After a 5-d incubation at 37°C and 0.5% CO_2_, the cell monolayers were visualized using naphthalene black. Viral titers were expressed as PFU per milliliter.

### Virus neutralization test

The virus neutralization test was performed as described previously (Širmarová et al., 2014), with several modifications. Briefly, monoclonal antibodies (T025, T028, T034, and T038) were diluted to 2.5 µg/ml in L-15 medium and then serially diluted 1:2 in 96-well plates. Diluted monoclonals were incubated with 50 PFU per well of TBEV-Hypr (sufficient to cause 90–95% cytolysis) for 90 min at 37°C. Thereafter, 5 × 10^4^ PS cells were added per well. After 4-d incubation (37°C), the cytopathic effect was monitored microscopically, and cell viability was measured using the Cell Counting Kit-8 (Dojindo Molecular Technologies) according to the manufacturer’s instructions. IC_50_ was calculated from two independent experiments done in octuplicates using GraphPad Prism (GraphPad Software; version 7.04).

Virus neutralization was also assayed using a fluorescence-based virus neutralization test. Monoclonal antibodies (T025, T028, T034, and T038; 10–1074 was used as an isotype control) were incubated with TBEV, and PS cells were infected as described above. The cells were incubated for 48 h at 37°C. The cell monolayers were fixed with cold acetone/methanol (1:1), blocked with 10% FBS, and incubated with mouse anti-flavivirus antibody (Sigma-Aldrich; 1:250 dilution, clone D1-4G2-4-15; catalog no. MAB10216). After washing, the cells were labeled with secondary goat anti-mouse antibody conjugated to FITC (Sigma-Aldrich; diluted 1:500, catalog no. AP181F) and counterstained with DAPI (diluted to 1 µg/ml) to visualize cell nuclei. The fluorescence signal was recorded with an Olympus IX71 epifluorescence microscope and processed by ImageJ software.

### Statistical analyses

Data were analyzed using Mann–Whitney tests or ANOVA and Tukey’s multiple comparison tests as specified and comparison of survival curves was analyzed by log-rank (Mantel–Cox) test, calculated in GraphPad Prism (version 8.4.3). P values < 0.05 were considered significant.

### Crystallization, structure determination, and refinement

Complexes for crystallization were produced by mixing Fab and antigen at a 1:1 molar ratio and incubating at room temperature for 1–2 h. Crystals of T025 Fab–TBEV^WE^ EDIII-His-Avitag complex (space group P2_1_; a = 55.5 Å, b = 66.7 Å, c = 91.2 Å, α = 90°, β = 94.6°, γ = 90°; one complex per asymmetric unit) were obtained by combining 0.2 µl crystallization complex with 0.2 µl of 0.1 M sodium citrate tribasic dihydrate, pH 5.0, 10% PEG 6000 in sitting drops at 22°C. Crystals of T025 Fab–TBEV^FE^ EDIII-His-Avitag complex (space group P2_1_2_1_2; a = 56.96 Å, b = 69.72 Å, c = 180.20 Å, α = 90°, β = 90°, γ = 90°; one complex per asymmetric unit) were obtained by combining 0.2 µl crystallization complex with 0.2 µl of 0.1 M sodium citrate tribasic dihydrate, pH 5.0, 10% PEG 6000 in sitting drops at 22°C. Crystals of T025 Fab–TBEV^Si^ EDIII complex (space group P2_1_; a = 55.4 Å, b = 67.2 Å, c = 91.2 Å, α = 90°, β = 94.8°, γ = 90°; one complex per asymmetric unit) were obtained by combining 0.2 µl crystallization complex with 0.2 µl of 5% (±)-2-methyl-2,4-pentanediol, 0.1 M Hepes, pH 7.5, 10% PEG 10,000 in sitting drops at 22°C. Crystals were cryoprotected with 25% glycerol before being cryopreserved in liquid nitrogen.

X-ray diffraction data were collected at Stanford Synchrotron Radiation Lightsource Beamline 12–2 using a Dectris Pilatus 6M detector. The data were integrated using Mosflm (Battye et al., 2011) and scaled using CCP4 (Winn et al., 2011). The T025-TBEV^WE^ EDIII complex structure was solved by molecular replacement using the V_H_V_L_ domains from PDB accession no. 2GHW, the C_H_C_L_ domains from PDB accession no. 4OGX, and TBEV EDIII from PDB accession no. 6J5F as search models in PHASER (McCoy et al., 2007). The model was refined to 2.24 Å resolution using an iterative approach involving refinement in Phenix (Adams et al., 2010) and manual rebuilding into a simulated annealed composite omit map using Coot (Emsley and Cowtan, 2004). Residues that were disordered and not included in the model were HC residues 131–132, 214–219 and the 6XHis tag; residue 214 of the LC; and residues 299–302, 397, and the 6XHis tag and Avi tag of the TBEV^WE^ EDIII domain. The T025-TBEV^FE^ EDIII and T025-TBEV^Si^ EDIII complex structures were solved similarly using the partially refined T025-TBEV^WE^ EDIII structure as the molecular replacement model. The T025-TBEV^FE^ EDIII model was refined to 2.35 Å resolution, and the T025-TBEV^Si^ EDIII model was refined to 1.86 Å resolution using the iterative approach described for T025-TBEV^WE^ EDIII. The Kabat numbering scheme was used for Fab numbering. Structures were superimposed, RMSDs were calculated, and figures were generated using PyMOL. Buried surface areas and hydrogen bonds were determined using PDBePISA (Krissinel and Henrick, 2007). Fab-antigen contact residues were identified as residues in which any atom is within 4 Å of an atom on the other protein. The distance and geometry criteria used for assigning hydrogen bonds were a distance of <4.0 Å and a hydrogen bond angle of 90–270°. The maximum distance allowed for a van der Waals interaction was 4.0 Å.

### Animal ethics statement

The research complied with all relevant European Union guidelines for work with animals and was in accordance with Czech national law guidelines on the use of experimental animals and protection of animals against cruelty (Animal Welfare Act No. 246/1992 Coll.). The protocol was approved by the Committee on the Ethics of Animal Experimentation of the Institute of Parasitology and the Departmental Expert Committee for the Approval of Projects of Experiments on Animals of the Czech Academy of Sciences (permit no. 4253/2019).

### Mice and virus inoculation

Specific pathogen–free BALB/c mice were obtained from ENVIGO RMS. Sterilized pellet diet and water were supplied ad libitum. In all experiments, female mice aged 6–8 wk were used. Mice were housed in individually ventilated plastic cages (Techniplast) with wood-chip bedding, with a constant temperature of 22°C, a relative humidity of 65%, and under a 12-h light/dark cycle. Three mice per group were used in experiments. Mice were inoculated i.p. 1 d before or 1, 3, or 5 d after infection with monoclonal antibodies T025 or 10–1074 in 200 μl PBS and infected subcutaneously with 100 PFU TBEV-Hypr (propagated eight times in suckling mouse brains). Mice were monitored for symptoms and survival over time and euthanized when reaching a humane endpoint.

### Online supplemental material

Fig. S1 shows correlations between clinical or demographic data, donor serum RVP neutralization or EDIII binding profiles, and RVP neutralization curves for vaccinee serum. Fig. S2 includes the single-cell sorting strategy, somatic hypermutations, and CDR3 length analysis for individual donors and all donors grouped together, as well as hydrophobicity GRAVY scores. Fig. S3 shows VH, VK, and VL gene frequencies; CDR3 sequence logos; and examples of antibody genes and CDR3 sequences that are similar across donors. Fig. S4 includes antibody binding to TBEV^FE^ and TBEV^Si^ by ELISA, screening results for antibody binding and neutralization of related tick-borne flaviviruses, and neutralization curves for selected antibodies against related tick-borne flaviviruses. Fig. S5 shows overlay of T025 crystal structures with the three TBEV-lineage EDIIIs, the amino acid alignment of three TBEV-lineage EDIIIs, and contact residues between T025 and TBEV EDIII. Table S1 contains clinical and demographic data for infected and vaccinated donors. Table S2 includes all antibody sequences obtained from sorted donor samples. Table S3 includes the sequences of antibodies that are similar across donors. Table S4 contains the sequences of the monoclonal antibodies that were cloned and recombinantly expressed. Table S5 shows EC_50_ and IC_50_ values for all antibodies tested. Table S6 contains data collection and refinement statistics for the crystal structures.

## Supplementary Material

Table S1 lists clinical data from TBEV-infected and vaccinated donors.

Table S2 lists sequences of anti-TBEV-EDIII IgG antibodies.

Table S3 lists highly similar antibody sequences shared across individuals.

Table S4 lists sequences of cloned recombinant monoclonal antibodies.

Table S5 lists effective and inhibitory concentrations of recombinantly expressed monoclonal antibodies.

Table S6 lists data collection and refinement statistics for the crystal structures.
