# Supplementary material for: Broad and potent neutralizing human antibodies to tick-borne flaviviruses protect mice from disease
Source: J Exp Med. 2021 Apr 8;218(5):e20210236. doi: 10.1084/jem.20210236 (PMC8040517; doi:10.1084/jem.20210236)
Supplement: Table S6 — lists data collection and refinement statistics for the crystal structures. [file JEM_20210236_TableS6.docx]

Table S1.

| **Complex** | **T025-TBEV^WE^ EDIII** | **T025-TBEV^FE^ EDIII** | **T025-TBEV^Si^ EDIII** |
| --- | --- | --- | --- |
| **PDB Accession Code** | **7LSF** | **7LSE** | **7LSG** |
| **Data Collection** |  |  |  |
| Resolution Range (Å) | 90.91 - 2.24 (2.32 - 2.24) | 90.10 - 2.35 (2.43 - 2.35) | 90.18 - 1.86 (1.91 – 1.86) |
| Space group | P 1 21 1 | P 2 21 21 | P 1 21 1 |
| Cell dimensions |  |  |  |
| a, b, c (Å) | 55.54, 66.73, 91.21 | 56.96, 69.72, 180.20 | 55.44, 67.23, 91.16 |
| α, β, γ (º) | 90, 94.64, 90 | 90, 90, 90 | 90, 94.81, 90 |
| Total reflections | 185353 (17430) | 362021 (36332) | 320468 (21062) |
| Unique reflections | 31036 (2897) | 30383 (2950) | 53401 (3528) |
| Multiplicity | 6.0 (6.0) | 11.9 (12.3) | 6.0 (6.0) |
| Completeness (%) | 97.2 (98.0) | 99.2 (99.6) | 95.8 (98.1) |
| Mean I/σ(I) | 8.8 (2.6) | 8.6 (3.1) | 8.5 (1.6) |
| Wilson B-factor (Å^2^) | 32.30 | 31.77 | 26.53 |
| R_merge_ | 0.117 (0.613) | 0.193 (0.861) | 0.107 (0.995) |
| R_pim_ | 0.078 (0.410) | 0.082 (0.365) | 0.071 (0.483) |
| CC_1/2_ | 0.994 (0.838) | 0.994 (0.853) | 0.995 (0.722) |
|  |  |  |  |
| **Refinement** |  |  |  |
| R_work_/R_free_ | 18.9% / 22.7% | 17.3% / 21.1% | 18.7% / 21.1% |
| Number of atoms | 4127 | 4169 | 4306 |
| Protein atoms | 4000 | 3961 | 3981 |
| Ligand atoms | 8 | 6 | 0 |
| Solvent atoms | 119 | 202 | 325 |
| Protein residues | 526 | 519 | 522 |
| RMS (bonds) (Å) | 0.007 | 0.008 | 0.017 |
| RMS (angles) (˚) | 0.95 | 0.95 | 1.44 |
| Ramachandran favored (%) | 97.1 | 97.9 | 96.7 |
| Ramachandran allowed (%) | 2.7 | 2.0 | 3.1 |
| Ramachandran outliers (%) | 0.2 | 0.2 | 0.2 |
| Clashscore | 2.7 | 2.9 | 4.1 |
| Average B-factor (Å^2^) | 36.97 | 37.90 | 34.93 |
| Protein atoms | 37.09 | 37.94 | 34.65 |
| Ligand atoms | 30.48 | 37.11 |  |
| Solvent atoms | 33.31 | 37.25 | 38.41 |
| Number of TLS groups | 13 | 16 | 15 |

Statistics for the highest-resolution shell are shown in parentheses.
